# The Elizabeth River Story: A Case Study in Evolutionary Toxicology

**DOI:** 10.1080/15320383.2015.1074841

**Published:** 2015-10-27

**Authors:** Richard T. Di Giulio, Bryan W. Clark

**Affiliations:** ^a^ Nicholas School of the Environment, Duke University, Durham, North Carolina, USA; ^b^ U.S. Environmental Protection Agency, Atlantic Ecology Division, National Health & Environmental Effects Research Laboratory, Office of Research and Development, Narragansett, Rhode Island, USA

## Abstract

The Elizabeth River system is an estuary in southeastern Virginia, surrounded by the towns of Chesapeake, Norfolk, Portsmouth, and Virginia Beach. The river has played important roles in U.S. history and has been the location of various military and industrial activities. These activities have been the source of chemical contamination in this aquatic system. Important industries, until the 1990s, included wood treatment plants that used creosote, an oil-derived product that is rich in polycyclic aromatic hydrocarbons (PAH). These plants left a legacy of PAH pollution in the river, and in particular Atlantic Wood Industries is a designated Superfund site now undergoing remediation. Numerous studies examined the distribution of PAH in the river and impacts on resident fauna. This review focuses on how a small estuarine fish with a limited home range, *Fundulus heteroclitus* (Atlantic killifish or mummichog), has responded to this pollution. While in certain areas of the river this species has clearly been impacted, as evidenced by elevated rates of liver cancer, some subpopulations, notably the one associated with the Atlantic Wood Industries site, displayed a remarkable ability to resist the marked effects PAH have on the embryonic development of fish. This review provides evidence of how pollutants have acted as evolutionary agents, causing changes in ecosystems potentially lasting longer than the pollutants themselves. Mechanisms underlying this evolved resistance, as well as mechanisms underlying the effects of PAH on embryonic development, are also described. The review concludes with a description of ongoing and promising efforts to restore this historic American river.

Much of the research concerned with ecological effects of environmental pollution in natural systems has involved lab dose-response studies to determine environmental concentrations that produce negative impacts on variables such as growth, reproduction, and survival in representative or model species that may parlay into population-level effects. Field studies that directly assess population condition, and measure sublethal effects (biomarkers) that may help inform population level effects, also play important roles. Further, studies addressing underlying mechanisms of toxicities are also useful. In addition to undergirding biomarkers, mechanistic studies inform extrapolations across chemicals in the environment and species that are not feasibly tested directly. These approaches have contributed significantly to our ability (1) to set reasonable criteria for chemicals in the environment, (2) to perform meaningful risk assessments, and (3) generally to protect the environment from chemical contamination.

However, an aspect of long-term environmental pollution that these approaches have limited capacity to address is that of adaptations by exposed organisms over multiple generations that improve the abilities of populations to thrive in polluted ecosystems. Of particular importance in this regard is the phenomenon of pollution-driven genetic adaptation, that is, the potential for pollution to act as a significant selection pressure potentially driving evolution. This phenomenon of “evolutionary ecotoxicology” has important ramifications for environmental science and management, including conservation biology, elucidation of fitness costs, and environmental risk assessment and remediation. For example, a site-specific risk assessment based upon an analysis of a population or community that evolved to resist pollution might lead the assessor to underestimate risks by not taking into account fitness costs, reduced genetic diversity, or other unknown consequences.

This review concerns a particular example, or case study, of this phenomenon, stressing biology but including historical, cultural, and management aspects. This story centers on the impacts of a ubiquitous class of environmental pollutants (polycyclic aromatic hydrocarbons [PAH]) on an ecologically important species of fish widespread along the Atlantic seaboard of North America (*Fundulus heteroclitus*, often referred to as mummichog or Atlantic killifish) in an estuary of great historical and commercial importance to the United States, the Elizabeth River, Virginia.

## THE ELIZABETH RIVER

### History

**FIGURE 1.**
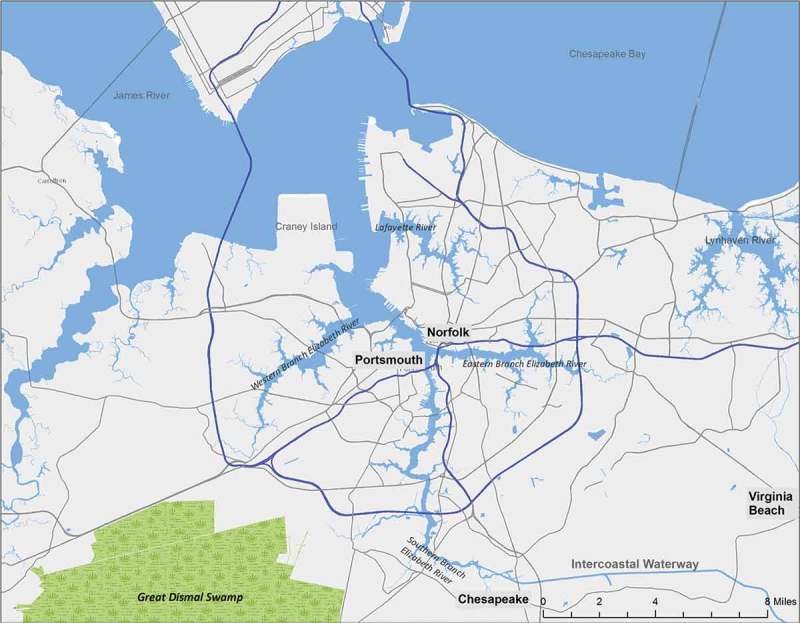
The Elizabeth River region, including major towns, connections to the James River and the Chesapeake Bay to the north, and to the Intracoastal Waterway and Dismal Swamp to the south.

The Elizabeth River is a tidal estuary in southeastern Virginia (Figure 1). This river consists of the Western, Eastern, and Southern Branches and the Lafayette River that flow through the towns of Chesapeake, Norfolk, Portsmouth, and Virginia Beach—important communities in the Hampton Roads region of Tidewater Virginia. The Southern Branch is the largest, flowing south to north, connecting to the Atlantic Intracoastal Waterway (ICW) near Great Dismal Swamp (which partially feeds the Southern and Western Branches) at its southern origin. After confluence of three branches, the river enters the James River near the mouth of the Chesapeake Bay, approximately 30 km north of its connection with the ICW (Figure 1). A detailed and fascinating history of the Elizabeth River was written by Amy Waters Yarsinske (*The Elizabeth River*, 2007, The History Press, Charleston, SC) and is the primary source of the following historical information.

Captain John Smith was involved in early explorations (circa 1605) of the river; he was sent by King James I of England to explore the Chesapeake Bay in order to establish a harbor for subsequent commerce. Around that time (exact date is uncertain), the river was named for Princess Elizabeth Stuart, the daughter of King James I and sister of Prince Henry and Prince Charles for whom early colonists named the capes at the entrance to the Chesapeake Bay. Following these early explorations, a major port was established on the river, near the mouth of the Chesapeake Bay; it is said to be at present the largest natural harbor in the world. Due to the existence of this harbor, its location on the Eastern Seaboard, and the abundance of natural resources in the area, the Elizabeth River played important roles in American history, particularly in the context of commerce and military activities.

In the late 18th century, the river was prominent in trade with Europe, Great Britain, and the East Indies for various commodities, including Indian corn, lumber, tobacco, naval stores, and rum (which was distilled in the Hampton Roads area). Prior to the Revolutionary War, a Scotsman, Andrew Sprowel, established the Gosport Shipyard on the Southern Branch, just below Portsmouth; the shipyard was established as a British government facility in 1767. In 1776, Norfolk and Portsmouth were bombarded by the British; this sparked reprisals resulting in confiscation of the shipyard by the Commonwealth of Virginia. Subsequently, numerous battles took place on the river during the Revolutionary War. The federal government leased the Gosport Shipyard after the U.S. Navy was created in 1794; 7 years later, in 1801, the government purchased the land and the facility’s name was changed to the Norfolk Naval Shipyard in 1862. The shipyard played a major role in building ships for the U.S. Navy during the War of 1812, the Civil War, and World Wars I and II. During World War II the Norfolk Naval Shipyard also served as the primary location for ship repairs—totaling 6,850 vessels.

### Physical Changes and Pollution

While commercial and military activities utilizing the Elizabeth River provided numerous benefits for the region and country, they have also had negative impacts on the health of this estuary. Perhaps the most dramatic effect that occurred during early European settlement and subsequent development was the clearing of old growth forests in the region, including riparian habitats lining the river’s branches. During the 1800s the river was dredged to roughly double its depth and filled in to about two-thirds of its original width, resulting in the loss of wetlands and shallows that provide critical habitat for many estuarine organisms. During the 19th and 20th centuries, the area’s growth in terms of human population, industrialization, naval activities, and shipping contributed to substantial pollution of this relatively poorly flushed estuary. Nutrients and bacteria largely associated with municipal effluents, pesticides in storm sewer runoff, heavy metals and polychlorinated biphenyls (PCB) from various industries, and creosote from wood treatment facilities are among the pollutants that generated the greatest concern, a concern that really materialized in the latter part of the 20th century. In a 1976 report required by the U.S. Environmental Protection Agency (EPA) and the U.S. Congress, the Virginia State Water Control Board called the Elizabeth River one of the worst water pollution problems in the state (Virginia State Water Control Board 1976). In 1993, the U.S. EPA listed the river as one of three regions of concern in the Chesapeake Bay due to chemical contamination (U.S. EPA, 1994); the other two were Baltimore Harbor and the Anacostia River (in Maryland and Washington, DC).

**FIGURE 2.**
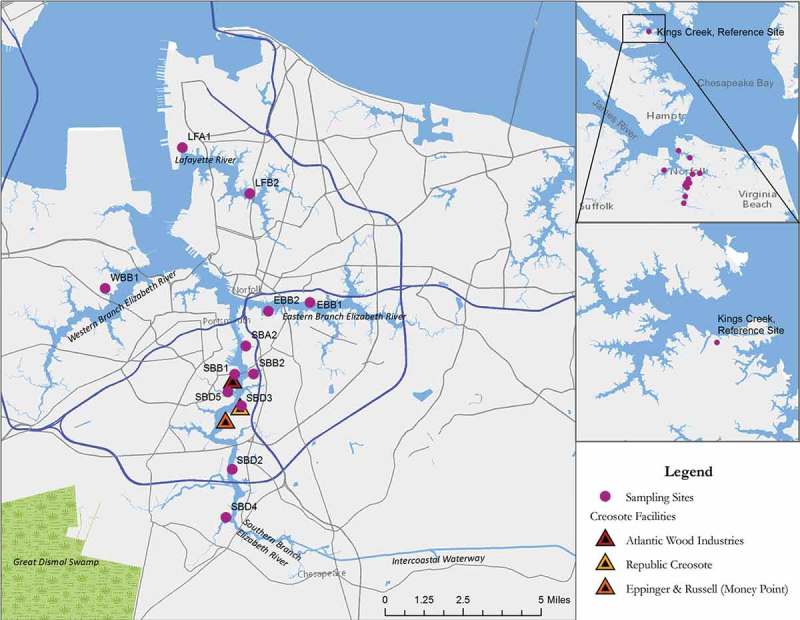
Locations of wood treatment facilities and sediment sampling sites in the Elizabeth and Lafayette rivers.

Among the dominant sources of pollution in the Elizabeth River, and of particular relevance to this story, have been wood treatment facilities, an industry that flourished in the area due to abundant timber resources, demand for wood products such as docks and railroad ties resistant to decay, and an extensive rail and water transportation infrastructure for shipping these and other products (Yarsinke 2007). Creosote derived from coal tar was patented and developed for wood treatment in the 1830s and rapidly became the wood treatment of choice for protecting wood from marine wood borers (e.g., *Teredo* spp. and *Limnoria* spp.) and other sources of wood decay (Nicholas 1973); it remains the most widely used wood preservative today (http://www.atsdr.cdc.gov/toxprofiles/tp.asp?id=66&tid=18). As its name implies, this form of creosote is derived from the distillation of coal, a commodity of great importance in Tidewater commerce. In the late 19th century Norfolk became the largest coal exporting port in the United States (Foreso et al. 1985), a position it holds through this day (http://www.eia.gov/todayinenergy/detail.cfm?id=3830). It is unclear how many wood treatment facilities operated in the Elizabeth River system. However, three major plants employing primarily creosote operated for the bulk of the 20th century, all in the Southern Branch—Atlantic Wood Industries, Republic Creosoting, and Eppinger and Russell (Figure 2) (Merrill and Wade 1985). All ceased operations in the latter part of the century. Atlantic Wood Industries was the last to close, in 1990, the same year it was placed on the National Priorities List (for Superfund sites); it is the only wood treatment plant on the Elizabeth to be so designated.

The chemistry of coal-derived creosote is dominated by PAH, which comprise the class of chemical contaminants that have been, and continue to be, of greatest concern in the Elizabeth River (Clark et al. 2013; Huggett, Bender, and Unger 1987; U.S. EPA 1994). In addition to creosote associated with wood treatment, other sources of PAH contamination in the river include coal and petroleum storage and transport facilities and shipbuilding and repair activities (Walker, Dickhut, and Chisholm-Brause 2004). The toxicological effect of PAH that has by far received the most attention and that underlies human health risk assessments is cancer (Luch 2005). Other important effects demonstrated by animal studies include suppression of the immune system (Kaplan et al. 2013) and perturbed development of the cardiovascular system (Billiard et al. 2008). As discussed in the following sections, all of these effects were observed in Elizabeth River killifish and/or in killifish exposed in lab studies to extracts of Elizabeth River sediments and to PAH present in the sediments. Killifish are a useful model to evaluate effects on other biota in this system, the majority of which are less amenable to field and lab studies.

## OVERVIEW OF ELIZABETH RIVER PAH CONTAMINATION AND BIOLOGICAL IMPACTS

### Polycyclic Aromatic Hydrocarbons in Sediments

The earliest published report we are aware of that addressed PAH pollution in the Elizabeth River is by Merrill and Wade (1985). This study included sediment analyses from five sites in the Southern Branch, including sites near the three wood treatment facilities noted earlier. An important conclusion of their study was that creosote was the major contributor of priority (i.e., carcinogenic) PAH in the river. A report shortly thereafter by Bieri et al. (1986) appears to comprise the first comprehensive analysis of PAH in the Elizabeth River system. This study measured PAH in sediment samples from 28 sites in the river, including sites from all three branches as well as the main stem downstream of the confluence of the branches. The highest PAH concentrations were observed in the vicinity of the wood treatment sites, including a maximum of 170 ppm for the sum of 14 pyrogenic PAH measured. (Note: All PAH concentrations in this review are expressed on a dry weight basis, as cited in the papers.) Bieri et al. (1986) concluded that creosote spills from wood treatment facilities were the likely source of these PAH. Moreover, a comparison made with data from four other PAH hotspots in the United States indicated that the Elizabeth River sediments contained the highest maximum PAH concentrations among these five sites.

Several studies published since Bieri et al. (1986) confirmed the high level of PAH contamination, the importance of wood treatment facilities in the Southern Branch, and added additional information concerning PAH dynamics in the system and impacts on biota in the estuary. Based on data collected in 1983 from 14 sites encompassing approximately 14 km in the main stem and Southern Branch, Huggett, Bender, and Unger (1987) concluded that the Elizabeth River was the most polluted estuary in Virginia, and that PAH emissions from wood treatment plants were the primary source of this pollution. This study also observed correlations between higher sediment PAH concentrations at different sites and higher tissue concentrations of PAH in deployed oysters, reduced fish biomass, decreased numbers of individual fish, and an increased occurrence of gross abnormalities in fish (fin erosion and eye lens cataracts). Huggett et al. (1992) summarized data from sediment surveys and fish trawls performed during the period of 1980–1989 and covering the entire 28 km of the subestuary (main stem and Southern Branch). Sediment chemistry again indicated a general increase in PAH concentrations as one moved from the mouth of the river to upstream sites near the wood treatment plants. A maximum total PAH concentration of 15 g/kg (1.5% or 15,000 ppm) was observed in sediments adjacent to a wood treatment facility. Results from the fish trawls also noted associations between sediment PAH concentrations and various lesions, similar to those reported by Huggett, Bender, and Unger (1987).

Walker and Dickhut (2001) used isomer ratios of selected PAH and molecular weight fractions to track PAH sources in the river (main stem and Southern Branch), using samples collected in 1999. Data showed that creosote from wood treatment plants was the predominant source, but that coal and/or coal gasification plants were also important in some areas. They noted interesting differences between the two plants that were focused on—Eppinger and Russell, and Atlantic Wood. Sediments adjoining Eppinger and Russell displayed higher total PAH concentrations (approximately 10× for maximal values) than those associated with Atlantic Wood. However, Atlantic Wood samples were more enriched in high-molecular-weight compounds (more associated with cancer than low-molecular-weight PAH). It is also noteworthy that despite the lower levels of PAH near Atlantic Wood, evidence indicated that this facility was a more important contributor of PAH into the main channel of the river, perhaps due to differences in hydrology. Similar conclusions were made by a subsequent report by Walker et al. (2005) that incorporated principal component analysis into the data analysis. It was also noted that historical and/or current coal transport and use were likely important sources of PAH at some sites in the river.

**FIGURE 3.**
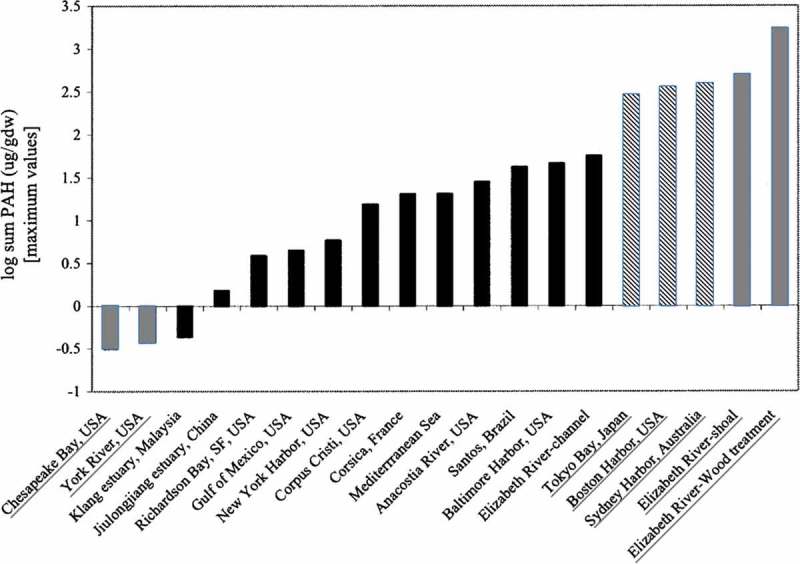
Concentrations of total PAHs in sediments from locations around the world. The highest value, “Elizabeth River—Wood Treatment,” is for sediment sampled adjacent to the Eppinger and Russell facility. From Walker, Dickhut, and Chisholm-Brause (2004); reprinted with permission from John Wiley and Sons.

A related report by Walker, Dickhut, and Chisholm-Brause (2004) involved the analysis of sediment samples collected in 1998–1999 (perhaps overlapping samples from the studies already described), including samples from near the mouth of the river (near Craney Island) to 22 km south of the mouth (which is several kilometers south of the wood treatment facilities; Figure 2). Again, the highest total PAH concentrations were found in samples for shoal areas near Atlantic Wood (740 ppm maximum) and Eppinger and Russell (1730 ppm maximum). The highest level observed in the main stem (just north and downstream of the confluence of the Southern and Eastern Branches) was 52 ppm, and was suspected to be due to a coal gasification plant. Included in this study was a comparison of these data with findings collected in 1983 (Huggett, Bender, and Unger 1987), about 15 years earlier. Sediment concentrations for most of the 10 PAH compared had declined at most sites, with calculated half-lives from 5 to 25 years. However, no significant decreases occurred for most PAH at 4 of 13 sites compared. At one site (between Eppinger and Russell and Atlantic Wood), increases were noted for three PAH with no change in the remaining 7. Notably, higher-molecular-weight PAH, including carcinogenic compounds, generally displayed slower removal rates. Walker, Dickhut, and Chisholm-Brause (2004) also compared their Elizabeth River findings with data from 16 other sites globally, including several PAH hotspots. Total PAH in shoal areas near wood treatment facilities in the Elizabeth River (740–1730 ppm) exceeded the highest levels reported for other contaminated sites including Tokyo Bay (˜300 ppm), Sidney Harbor (˜400 ppm), and Boston Harbor (˜400 ppm). Non-source-specific areas in the river’s channel (˜60 ppm) were below these three locations, but greater than most other sites included in the comparison. For example, PAH concentrations in the nearby York River were ˜3 ppm (Figure 3).

Two reports by Vogelbein and Unger (2003; 2008) to the Virginia Department of Environmental Quality provided extensive data for various sites in the Elizabeth River system, including the three branches, the Lafayette River, and sites proximate to former wood treatment facilities. Average total PAH concentrations for selected sites (Figure 2) from the 2003 and 2008 reports are included in Table 1. Note that samples for the 2003 and 2008 reports were collected in 2001 and 2007, respectively, and the 2008 report added several sites in the vicinity of Money Point. The Money Point area includes sites that were impacted by the Eppinger and Russell facility (on land now owned by Hess Corporation) that are presently undergoing remediation. These reports also include observations of fish pathology surveys that are described later. Sediment data (Table 1) demonstrate continued highly elevated PAH concentrations near wood treatment facilities years to decades after cessation of plant operations. Moderate to low levels were reported in the Southern Branch downstream of the facilities, in the Western and Eastern Branches, and in the Lafayette River. Generally low concentrations were observed upstream of the facilities in the Southern Branch. Sediment PAH data provided by Clark et al. (2013) for samples collected in 2008 are not directly comparable due to differences in sampling sites and chemical analyses, but demonstrate a similar spatial distribution. It is also noteworthy that collectively data from Vogelbein and Unger (2003, 2008) did not indicate widespread declines of PAH levels in this system over this time frame. There are apparently no more recent sediment data available.

**TABLE 1.** Total Concentrations of Select Polycyclic Aromatic Hydrocarbons (PAHs) in Sediment (ppb or ng/g Dry Weight) From Study Sites Sampled in 2001 and 2007 in the Elizabeth River, Virginia

Site ID (Figure 2 ID)	2001 (ppb)^a^	2007 (ppb)^b^
Lafayette River-A1 (LFA1)	5,596	24,678
Lafayette River–B2 (LFB2)	706	1,978
Western Branch–B1 (WBB1)	276	1,339
Eastern Branch–B1 (EBB1)	917	3,004
Eastern Branch–B2 (EBB2)	52,402	24,398
Southern Branch–A2 (SBA2)	25,295	23,730
Southern Branch–B2 (SBB2) (Scuffeltown Creek)	13,562	26,375
Southern Branch–B1 (SBB1) (Atlantic Wood Industries site)	490,815	383,186
Southern Branch–D5 (SBD5)	4,428	5,850
Southern Branch–D3 (SBD3) (Republic Creosote site)	144,931	113,885
Southern Branch–D2 (SBD2)	190	2,226
Southern Branch–D4 (SBD4)	208	736

^a^Site IDs and PAH data based on Vogelbein and Unger (2003).

^b^Site IDs and PAH data based on Vogelbein and Unger (2008).

### Biological Responses Associated With Pollution

In addition to these studies addressing chemical contamination of the Elizabeth River, numerous studies examined biological responses potentially associated with pollution in the river. The vast majority of investigations described in this section, as well as those assessing sediment chemistry described earlier, was carried out by scientists at the Virginia Institute of Marine Science (VIMS), a component of the College of William and Mary. The earliest reports of biological effects that were available are those by Hargis, Roberts, and Zwerner (1984) and Weeks and Warinner (1984). The former study involved exposures of spot (*Leiostomus xanthurus*) from an uncontaminated estuary (Ware River, VA) to sediments from a creosote-contaminated location in the Southern Branch of the Elizabeth River. Compared to control fish, exposed spot exhibited severe skin, fin, and gill erosion, reduced hematocrits, and pancreatic and liver lesions. Several other early field investigations also noted various internal and external lesions in a variety of species captured in the Elizabeth River (Huggett, Bender, and Unger 1987; 1992; Thiyagarajah, Zwerner, and Hargis 1989). A lab study by Sved, Roberts, and VanVeld (1997) exposed spot to sediment amended with low- or high-molecular-weight fractions of creosote (both PAH primarily) and concluded that the high-molecular-weight fraction accounted for lesions and mortality.

Several investigations addressed immune function in Elizabeth River animals. In a companion study to Hargis, Roberts, and Zwerner (1984), Weeks and Warinner (1984) collected from the Elizabeth and Ware rivers spot and hogchokers (*Trinectes maculatus*), from which they collected kidney macrophages and assessed phagocytosis. Phagocytic activity was reduced in both species from the Elizabeth River following exposures, but rebounded after several weeks in clean water. These observations were consistent with those of a subsequent paper (Faisal et al. 1991) that found suppressed phagocytic activity by leukocytes from killifish captured in the Elizabeth River compared to killifish from a reference site in the York River. Chu and Hale (1994) and Chu et al. (2002) examined the effects of Elizabeth River sediment extracts on several aspects of immune function in the eastern oyster (*Crassostrea virginica*), including susceptibility to the protozoan parasite *Perkinsus marinus* (dermo). These studies demonstrated enhanced disease expression in exposed oysters, which may be of importance in light of the long-standing concerns for this disease in the Chesapeake Bay region.

Perhaps the disease of greatest concern that is associated with chemical pollution, particularly by PAH, in the Elizabeth River is cancer. Killifish, particularly fish collected from near the Atlantic Wood Industries site, have been the focus of this phenomenon. The first report in this vein appears to be by Hargis et al. (1989), who reported a 2% incidence (among 398 fish necropsied) of exterior neoplasms, mainly in the mouth area. This also appears to be the earliest publication concerning pollution effects on Elizabeth River killifish, the focus of this review. Vogelbein et al. (1990) found that effects on liver tissue were more severe. Of the 60 killifish collected from the Atlantic Wood Industries site, 93% exhibited grossly visible liver lesions and 35% displayed frank hepatocellular neoplasms (i.e., liver cancer). Subsequently, Stine et al. (2004) provided detailed information concerning the nature of these lesions. Rose et al. (2000) observed highly elevated levels of hydrophobic DNA adducts in hematopoietic tissues (liver, kidney, spleen) and blood in killifish from this site, whereas none were detected in reference site fish. These adducts, likely derived from PAH, provide a plausible link between PAH exposures and cancer in these fish. Further, Vogelbein and Unger (2003, 2008) described findings that appear to be the most extensive and most recent surveys of liver pathologies in the Elizabeth River system. These reports include fish collected from 16 sites covering all three of the branches of the Elizabeth River and the Lafayette River. Evidence in the 2008 report indicated that overall the incidence of liver disease, including cancer, had not changed significantly from the previous survey, and that the environmental quality of the system remained significantly impaired. These studies support the utility of liver disease in killifish as an effective measure and bioindicator of estuarine contamination, at least for PAH.

In apparently the earliest report of biochemical effects, Roberts, Sved, and Felton (1987) measured activities of superoxide dismutase (SOD) and aryl hydrocarbon hydroxylase (AHH) in livers of spot, again collected over several months from the Elizabeth and Ware rivers. SOD is an antioxidant enzyme, while AHH was an early assay for cytochrome P-450 (CYP1A) activity, a key part of the aryl hydrocarbon receptor (AHR) pathway that is described later (Figure 5, shown later). For both activities, Roberts et al. (1987) noted significant but inconsistent elevations in Elizabeth River fish relative to Ware River fish. Using more advanced techniques for measuring CYP1A, a seminal paper by Van Veld and Westbrook (1995) examined killifish from several sites, including Atlantic Wood Industries, and showed that while field-collected fish had higher basal activities than reference-site fish, they were remarkably recalcitrant to enzyme induction by a potent inducer. This study and a VIMS master of arts thesis (Williams 1994) demonstrated the resistance of offspring of this same population to acute toxicity induced by Atlantic Wood Industries sediments, which was the original motivation for the subject of this review: pollution-driven evolution in Elizabeth River killifish.

## THE ATLANTIC KILLIFISH OR MUMMICHOG (*Fundulus heteroclitus*)

### Basic Biology

**FIGURE 4.**
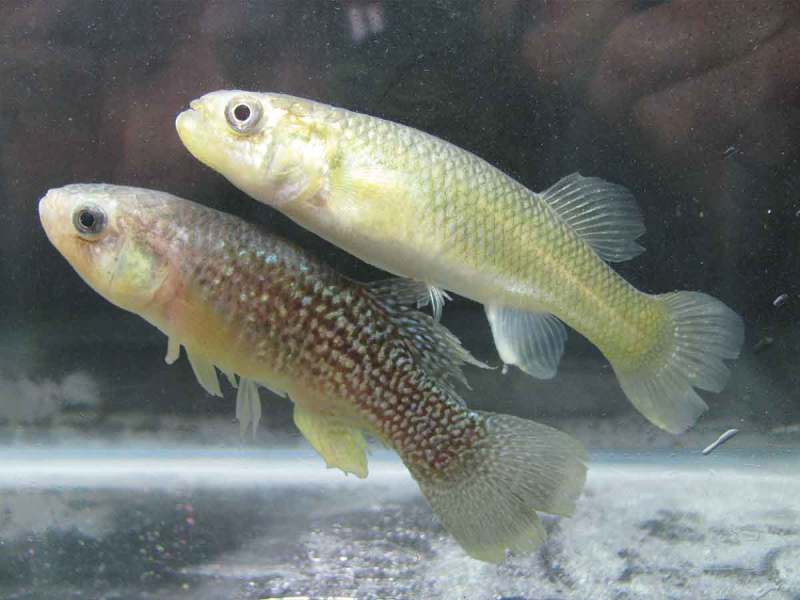
Adult female (above) and male (below) *Fundulus heteroclitus* (Atlantic killifish, mummichog).

The Atlantic killifish is a small teleost fish found in estuarine ecosystems along the Atlantic coast of North America from New Brunswick to Florida (Shute 1980). This species has a lifespan of about approximately 3–4 years, is sexually dimorphic, and reaches sexual maturity at less than 1 yr old (Abraham 1985) (Figure 4). It is among the most abundant intertidal fish and a major component of estuarine food webs (Yozzo et al. 1994; Teo and Able 2003a). Killifish are omnivorous feeders; reported food sources include plant detritus, macroalgae, grass shrimp, crabs, annelids, and other fish (Kneib 1986; Kneib and Stiven 1978; Allen et al. 1994; McMahon, Johnson, and Ambrose 2005; Able et al. 2007). In turn, killifish are prey for birds, fish, and invertebrates (Post 2008; Nemerson and Able 2003; Kneib 1982). In general, killifish spawn near the high tide line on a semilunar schedule concurrent with tidal changes (Taylor et al. 1979).

**FIGURE 5.**
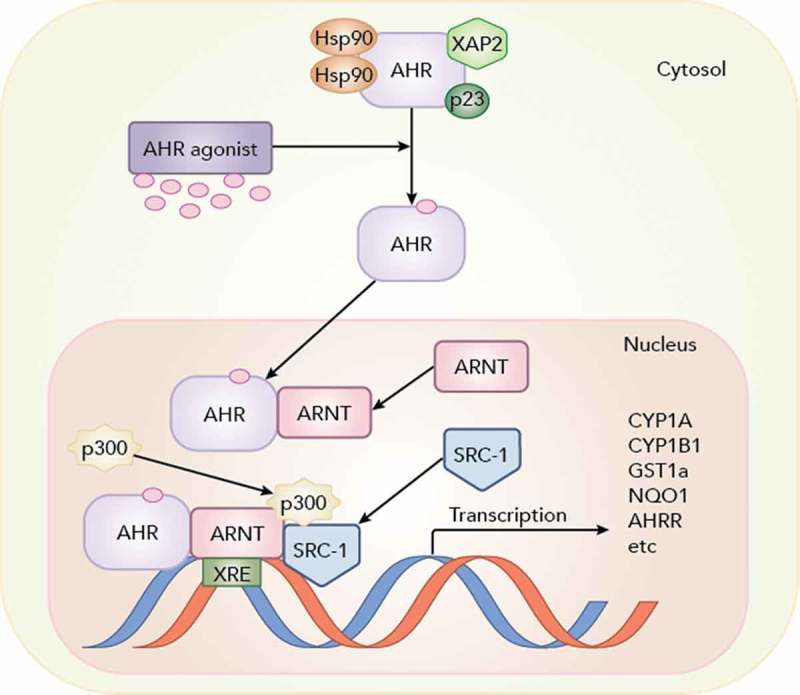
The aryl hydrocarbon receptor (AHR) pathway. The pathway is activated by binding of the AHR to ligands that include dioxin-like chemicals (DLCs) and some polycyclic aromatic hydrocarbons (PAHs). This allows for dimerization with the AHR nuclear transporter (ARNT) that forms the transcriptionally active complex that binds to xenobiotic response elements (XRE) and thereby upregulates the transcription of a number of proteins including enzymes involved in biotransformation, indicated here, as well as the AHR repressor (AHRR) that provides negative feedback of the pathway. Hsp90, XAP2, and p23 are chaperone proteins; SRC-1 and p300 are examples of co-regulator proteins involved in transcription. © McGraw-Hill Education. Reproduced by permission of McGraw-Hill Education. Permission to reuse must be obtained from the rightsholder.

In addition to their important role in estuarine ecosystems, killifish have a number of attributes that make them a unique and popular research species for environmental research (Burnett et al. 2007). Killifish are tolerant of significant variation in environmental conditions, including salinity, temperature, oxygen, and pH (Gonzalez, Mason, and Dunson 1989; Dunson, Fricano, and Sadinski 1993; Wood and Marshall 1994; Smith and Able 2003; Stierhoff, Targett, and Grecay 2003; Nordlie 2006). They are easy to capture and maintain, amenable to manual spawning, and highly fecund, with a single female able to produce up to several hundred eggs at once. Eggs (about 1–2 mm in diameter) have a transparent chorion that facilitates observation of development and stages of embryonic development and organogenesis that are well known (Armstrong and Child 1965). Despite wide distribution, individual killifish have relatively small home ranges (Lotrich 1975; Skinner et al. 2005) and are ideal for studying impacts of local contamination and other stressors (Mulvey et al. 2002, 2003; Burnett et al. 2007; Eisler 1986; Teo and Able 2003b, 2003a). Further, their tolerance to a wide variety of environmental conditions such as variations in salinity, dissolved oxygen, and temperature has made them an extremely useful model to investigate adaptation to environmental changes.

### Role of Killifish in Environmental Adaptation Research

##### Osmoregulation

Killifish have played an important role in the study of osmoregulation and continue to be an important model in this field. Tolerance to an extreme range of salinity from freshwater to hypersalinity as high as 120‰ (Griffith 1974), has made them a valuable tool for studying accommodation to both fresh and salt water, yielding critical contributions to the understanding of teleost osmoregulation (Evans 2008; Evans, Piermarini, and Choe 2005; Burnett et al. 2007). Of particular importance was their contribution to the development of the model of NaCl excretion in what are now known as mitochondria rich cells (MRC) but were formerly known as chloride cells. Karnaky and coworkers (1976) localized the Na^+^,K^+^-ATPase responsible for Na^+^ excretion in killifish MRC and also showed the role of the same cells in Cl- excretion (Degnan, Karnaky, and Zadunaisky 1977; Karnaky, Degnan, and Zadunaisky 1977). Later, Marshall et al. (1995) identified the channel responsible for Cl- excretion, and Singer et al. (1998) cloned the channel, which is a homologue of the mammalian cystic fibrosis transmembrane conductance regulator. These discoveries helped to complete the model of NaCl secretion in marine teleosts that is still used today.

##### Hypoxia response

Killifish thrive in areas with variable and often low dissolved oxygen (O_2_) concentrations. A number of studies showed that killifish survive exposure to dissolved O_2_ levels as low as 1 mg/L (Stierhoff, Targett, and Grecay 2003; Smith and Able 2003; Wannamaker and Rice 2000). In fact, killifish may be more tolerant of hypoxia than other common estuarine fish, including *Cyprinodon variegatus* (sheepshead minnow), *Lucania parva* (rainwater killifish), and *Menidia beryllina* (inland silverside) (Smith and Able 2003). In addition, unlike other estuarine species, killifish did not exhibit avoidance of water with low dissolved O_2_ in lab choice experiments (Wannamaker and Rice 2000). One important behavior that helps killifish greatly in low O_2_ conditions is utilization of the more air-saturated surface of the water, which is known as aquatic surface respiration (ASR) (Stierhoff, Targett, and Grecay 2003; Wannamaker and Rice 2000; Greaney et al. 1980).

To cope with chronic exposure to low O_2_ conditions, killifish undergo a number of physiological changes. Their hematocrit and hemoglobin O_2_ affinity increase to augment the O_2_-binding capacity in blood (Stierhoff, Targett, and Grecay 2003; Greaney et al. 1980; Greaney and Powers 1977). In addition, killifish shift toward anaerobic metabolism, via glycolysis, to supplement energy production during O_2_ tension. This is reflected in increased blood and tissue concentrations of lactate observed in this species under O_2_ stress (Cochran and Burnett 1996; Greaney et al. 1980). In addition, increases in the activity of multiple glycolytic enzymes have been documented in killifish exposed to chronic hypoxia (Kraemer and Schulte 2004; Greaney et al. 1980).

##### Temperature adaptation

In addition to tolerating a wide range of salinity and O_2_ conditions, killifish populations are distributed in habitats along a thermal gradient that covers approximately 12°C. This has resulted in adaptive differences between populations at the extremes of this range and made killifish an important model for study of molecular evolution and environmental adaptation (Schulte 2001). In a series of studies from the late 1970s through the early 1990s, Powers at Stanford University and colleagues investigated the mechanisms underlying this adaptation. Examination of genetic variation among killifish populations along this temperature gradient showed that there are distinct northern and southern genotypes of killifish (Bernardi, Sordino, and Powers 1993). In particular, research focused on the glycolytic enzyme heart-type lactate dehydrogenase (Ldh-B), which was found to exist as two predominant allozymes that had significant differences in enzyme activity at different temperatures (Place and Powers 1979). Specifically, Ldh-B associated with northern populations exhibited a higher reaction rate at lower temperatures, whereas Ldh-B associated with southern populations exhibited a higher reaction rate at higher temperatures. In addition, northern populations possess significantly higher overall levels of Ldh-B transcription (Crawford and Powers 1989; 1992). Eventually, the observed differences in transcription were attributed to differences between populations in the sequence of the promoter region (Schulte, GomezChiarri, and Powers 1997; 2000). These findings were strongly correlated to whole organism performance tests. Individuals homozygous for the northern Ldh-B allele were found to swim faster at lower temperatures than individuals homozygous for the southern allele (DiMichele and Powers 1982).

##### Recent development as a molecular model

Because killifish successfully inhabit and tolerate a wide variety of environmental conditions, they have great potential for investigation of genome–environment interactions (Burnett et al. 2007). In addition to the advantages of studying wild populations that face significant daily variation in environmental stressors such as temperature, salinity, and dissolved O_2_, killifish may be compared to their diverse relatives in the family Fundulidae or to other popular fish models, such as zebrafish (*Danio rerio*). Further, the killifish genome has now been fully sequenced; it is currently being assembled and annotated, but is already available to the community in its current form (https://my.mdibl.org/display/FGP/Home). Utilizing the newly sequenced genome, resequencing of individuals from the major adapted killifish populations is underway in multiple labs to identify the genomic signatures of pollution adaptation. However, significant progress has already been made using transcriptomic and other molecular approaches to study pollutant responses in killifish.

Several studies used differential display or subtractive hybridization to examine gene expression in response to contaminants. Meyer et al. (2005) employed this approach to identify genes differentially expressed in PAH-tolerant and -susceptible populations. Suppressive subtractive hybridization was used to develop “fingerprints” of exposure to the PAH anthracene and pyrene (Peterson and Bain 2004; Roling, Bain, and Baldwin 2004) and to discern the impact of arsenic exposure on gene expression in offspring (Gonzalez et al. 2006). Other studies used cDNA arrays to investigate the effects of chromium, both in lab exposures and in fish from contaminated sites (Roling et al. 2006; 2007; Roling and Baldwin 2006). In addition, multiple researchers compared gene expression among populations from a series of polluted and reference sites; several of these studies included the Elizabeth River population and are discussed later.

Killifish microarrays have also been used to investigate evolution of regulation of gene expression (Crawford and Oleksiak 2007; Whitehead and Crawford 2006b). Studies in killifish enable investigators to compare variation in expression among individuals within distinct populations, between populations, and across related Fundulidae. Several studies demonstrated a high degree of variation in gene expression among and within populations and between taxa that is likely to be evolutionarily important (Whitehead and Crawford 2006a; Oleksiak, Churchill, and Crawford 2002; 2005).

Several transgenesis techniques have been employed in killifish, and their use is likely to expand with the sequencing and ongoing annotation of the genome. The feasibility of transgenesis was demonstrated by Winn, Vanbeneden, and Burkhart (1995), who developed a model of in vivo mutagenesis in killifish. Further, morpholino gene knockdown was recently adapted for use in killifish. Morpholinos are antisense oligonucleotides that may be used to transiently block translation of targeted mRNAs. They are particularly useful for studying gene function during development in fish. In killifish, morpholino knockdown of CYP1A was used to validate the technique and investigate the synergistic effects generated by AHR agonists and CYP1A inhibition (Matson et al. 2008). Morpholino knockdown was also used to demonstrate which of the two known killifish AHR mediates PAH toxicity during development (Clark et al. 2010). The AHR pathway is illustrated and described in Figure 5 directly after this section, and the subject of AHR-mediated toxicity is expanded upon later. In addition, morpholinos have been used to efficiently block translation of serum glucocorticoid kinase 1 and mitogen activated protein kinase 14–1 in studies of seawater acclimation (Notch et al. 2011; 2012) . Following the completion of genome annotation and assembly, there appear to be no barriers to the extensive use of these and other molecular methods in killifish.

## THE POLLUTION-ADAPTED PHENOTYPE

### Discovery of PAH Resistance in Elizabeth River Killifish

As described previously, initial studies in and around the Atlantic Wood Industries Superfund Site on the Elizabeth River focused on severe acute toxicity affecting a wide variety of fish; data clearly showed that organisms in the estuary were highly impacted by creosote contamination. After the observation of neoplasia in killifish, further attention was focused on that particular species. Given their relatively nonmigratory nature, the Atlantic Wood killifish population appeared likely to be a stable, persistent population that was exhibiting tumors primarily due to long-term residence in a highly creosote-contaminated habitat. Despite the severity of tumorigenesis in killifish resident at this site, it appeared that Atlantic Wood killifish were somehow capable of circumventing many of the acute toxic effects seen in spot and other fish and managing to thrive despite highly elevated pollutant exposures. Studies in several labs addressed and are continuing to examine this remarkable degree of resistance, including the elucidation of underlying mechanisms.

Evidence of resistance of Elizabeth River killifish to effects of PAH was first published by Van Veld and Westbrook (1995). In this study, cytochrome P-4501A (CYP1A) response was compared in adult fish collected from the Atlantic Wood site and two reference sites (Wilson Creek and King’s Creek, both north of the mouth of the York River, in Gloucester County, VA). CYP1A is a major enzyme involved in Phase I, oxidative biotransformation of xenobiotics. It is highly inducible by exposure to a wide-range of chemicals, including PAH and other aryl hydrocarbons (Figure 5). As might be expected, CYP1A levels in freshly caught fish were elevated in Atlantic Wood fish in comparison to reference fish (5-fold in liver, 56-fold in gut). However, following intraperitoneal (ip) injection of the PAH 3-methylcholanthrene (3-MC), hepatic expression of CYP1A protein increased 418-fold in reference (King’s Creek) fish but was not significantly changed in Atlantic Wood fish. Van Veld and Westbrook (1995) also compared CYP1 activity following 3-MC injection using the ethoxyresorufin *O*-deethylase (EROD) assay, a fluorescence-based measurement of CYP1 catalysis, and EROD activity followed the same trend as CYP1A protein. Further, when each group was exposed to contaminated Elizabeth River sediments, hepatic CYP1A protein expression and EROD activity were markedly elevated in fish from the reference site compared to levels measured in fish from the Atlantic Wood site. In total, data demonstrated that fish from the Atlantic Wood site exhibited a remarkable recalcitrance to induction of CYP1A by PAH.

### Characterization of PAH Resistance in Elizabeth River Killifish

After identification of this striking resistance by Atlantic Wood killifish to one of the major biological responses to PAH exposure, greater attention was focused on characterizing the breadth of PAH resistance of Elizabeth River killifish. Due to the probability of close contact with contaminated sediments and potential for increased sensitivity in early life stages, effects on embryos were, and continue to be, of particular interest. Further, it is likely that heritable PAH adaptation is driven by acute toxicity and early-life-stage effects that might impact reproductive success, rather than chronic conditions such as cancer. Ownby et al. (2002) investigated early-life-stage toxicity induced by PAH-contaminated sediments in killifish embryos from populations inhabiting four sites on the Elizabeth River, including the Atlantic Wood site, and a reference population on the nearby York River. Sediment PAH concentrations detected among the Elizabeth River sites ranged from 3.9 ± 3.2 to 264 ± 115 mg/g (ppm, dry weight, sum of select PAH). Field-collected adults were manually spawned in the lab, and tolerance of their offspring to Atlantic Wood site sediment was compared. Embryos obtained from the reference population suffered from a variety of cardiac abnormalities, including tube hearts, reduced circulation, and pericardial swelling, but embryos from Atlantic Wood parents showed a dramatic and near-complete resistance to these effects. The observed cardiovascular defects were similar to the suite of deformities known as blue-sac disease produced by some halogenated aromatic hydrocarbons such as 3,3’,4,4’,5-pentachlorobiphenyl (PCB-126) and 2,3,7,8-tetrachlorodibenzodioxin (TCDD) (Peterson, Theobald, and Kimmel 1993). (These halogenated compounds that exert toxicity via agonism of the AHR are commonly referred to as “dioxin-like compounds” or DLC. Some PAH including several occurring in the Elizabeth River are also AHR agonists.) Ownby et al. (2002) also showed that embryos from other Elizabeth River populations showed an intermediate degree of tolerance to the same effects. The degrees of resistance were roughly associated with sediment PAH concentrations at the sites of collection, perhaps reflecting population exposure histories. In addition, Ownby et al. (2002) tested the heritability of tolerance by exposing F2 embryos obtained from lab-reared F1 adults. The F2 embryos exhibited a degree of tolerance similar to that exhibited by F1 embryos, indicating that resistance was heritable.

**FIGURE 6.**
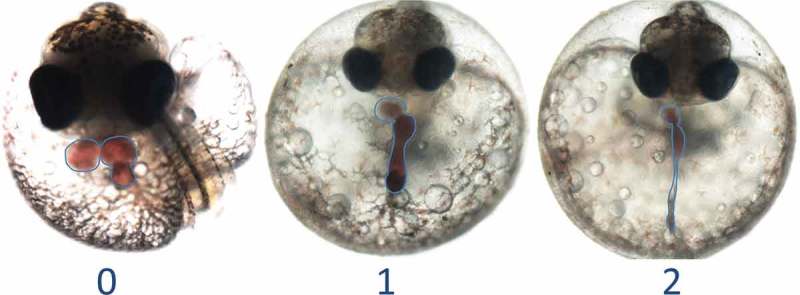
Effects of exposures to extracts of sediments from the Elizabeth River (Atlantic Wood Industries site) on the developing cardiovascular system in Atlantic killifish embryos. These embryos are offspring of adults collected from a reference site (King’s Creek). The “0” embryo shows a healthy 2-chambered heart (circled, just below eyes) and the bulbus arteriosis. The “1” and “2” embryos exhibit progressive malformation of the heart into what is referred to as a “stringy” or “tube” heart. These exposures do not produce this effect on offspring of adults collected from the Atlantic Wood site.

The resistance of Atlantic Wood killifish to PAH (Meyer, Nacci, and Di Giulio 2002; Meyer and Di Giulio 2003) and PCB-126 (Meyer and Di Giulio 2002) was further investigated. In addition to examining resistance to toxicity from contaminated sediments and PAH, these studies further determined the pattern and heritability of recalcitrant CYP1A response previously observed in adult Elizabeth River killifish by Van Veld and Westbrook (1995). Despite sediment contamination dominated by PAH and not DLC including PCBs, F1 Atlantic Wood embryos were highly resistant to cardiac teratogenesis and induction of CYP1 activity generated by PCB-126 exposure (Meyer and Di Giulio 2002). In fact, data indicated that both F1 and F2 Elizabeth River killifish were even more resistant to PCB-126 than adapted killifish from DLC-contaminated sites in Newark Bay, NJ, and New Bedford Harbor, MA (Nacci, Champlin, and Jayaraman 2010). Atlantic Wood F1and F2 embryos were also resistant to developmental abnormalities and mortality observed in reference embryos exposed to Elizabeth River sediment pore water (Meyer, Nacci, and Di Giulio 2002; Meyer and Di Giulio 2003). Typical effects on the developing cardiovascular system observed in reference embryos but not Atlantic Wood embryos are displayed in Figure 6. In addition, Atlantic Wood F1 embryos were recalcitrant to induction of CYP1A by the AHR agonist-type PAH 3-MC and β-naphthoflavone (BNF).

However, in some studies resistance was not consistently heritable. PCB-126-induced EROD activity in F3 embryos (F2 were not tested) returned to levels similar to those of reference embryos; at the highest doses tested, resistance by F3 embryos to the teratogenic effects of PCB-126 was intermediate between the highly resistant F1 embryos and susceptible reference embryos (Meyer and Di Giulio 2002). Similarly, recalcitrance to CYP induction was largely lost in F3 embryos and F2 larvae dosed with 3-MC, BNF, or sediment pore water (Meyer, Nacci, and Di Giulio 2002; Meyer and Di Giulio 2003). In addition, hepatic EROD activity in adult Atlantic Wood F2 and even F1 adults exposed to Elizabeth River sediments was found to be similar to that of adult reference fish. In contrast to the results of Ownby et al. (2002) and Nacci, Champlin, and Jayaraman (2010), these studies by Meyer and colleagues (2002) seemed to demonstrate that the resistance was heritable but not fully genetic, and it was proposed that this might be achieved through epigenetic regulation of CYP1A. However, Timme-Laragy et al. (2005) found no marked differences between methylation status of CpG sites in the CYP1A promoter of Elizabeth River and reference fish. Interestingly, hybrid embryos generated by crossing Atlantic Wood fish of either sex with reference fish demonstrated a BNF-induced EROD response intermediate between those of reference and Atlantic Wood embryos (Meyer, Nacci, and Di Giulio 2002). In a subsequent study, survival of hybrid larvae exposed to diluted Elizabeth River pore water was intermediate between that of Atlantic Wood and reference larvae (Meyer and Di Giulio 2003). In both cases, the response of the two hybrid lines was nearly indistinguishable, regardless of the sex of the parent from the Atlantic Wood population. These results seem to be more consistent with a hypothesis of genetically heritable resistance, transmitted by both male and female Atlantic Wood fish.

It is difficult to reconcile the results of Meyer and coworkers (2002) with those of Ownby et al. (2002) and Nacci, Champlin, and Jayaraman (2010), although it is notable that some data obtained by Meyer, Nacci, and Di Giulio (2002) supported a conclusion of genetic heritability, but others do not. Further contributing to this complex picture, Clark, Bone, and Di Giulio (2014) recently found that while F1 and F2 Atlantic Wood embryos were strongly resistant to teratogenesis induced by several PAH and PCB-126, their resistance to induction of CYP1A, CYP1B1, and CYP1C1 mRNA and EROD activity was reduced in F2 embryos, which suggested that only some aspects of the resistance were genetically inherited. In general, the strongest evidence for full genetic heritability has been observed for resistance to teratogenesis in embryos, while studies of heritable resistance in larvae and adults yielded mixed results. Further, the resistance to toxicity and the refractory CYP response seem to decrease with age or after generations of lab rearing, perhaps indicating that some components of the adaptation are life-stage specific or not genetically heritable. It is also possible that resistant fish are less fit under clean conditions, causing some lab populations to undergo “reverse” selection, yielding less resistant offspring in later generations (Meyer and Di Giulio 2003).

### Mechanisms of PAH Resistance in Elizabeth River Killifish

##### Phase II conjugation enzymes

Multiple studies addressed the mechanisms underlying adaptation of Elizabeth River *Fundulus* to acute toxicity and teratogenesis produced by PAH mixtures. Early in the study of the Atlantic Wood population, investigators discovered elevated expression of a number of xenobiotic metabolizing enzymes. Phase II enzymes, such as glutathione *S*-transferases (GST), act to conjugate xenobiotics, increasing their solubility and enhancing their excretion from the body. In an investigation of the hepatic lesions in Atlantic Wood killifish, Van Veld et al. (1991) noted that hepatic and intestinal GST activity was elevated three- to fourfold in Atlantic Wood fish with respect to reference fish. Armknecht, Kaattari, and Van Veld (1998) postulated that elevation in GST might contribute to the PAH resistance. They confirmed earlier findings (Van Veld et al. 1991) of elevated GST activity in Atlantic Wood fish and also a concomitant rise in protein levels. In both studies, GST elevation was found to be intermediate in fish from a site with intermediate levels of PAH, perhaps indicating that increased GST was a marker of exposure rather than an adaptive change. However, hepatic GST activity was not induced in reference fish fed a BNF-amended diet (Van Veld et al. 1991). Gaworecki, Rice, and Van Den Hurk (2004), observed elevated activity of the Phase II enzyme UDP-glucuronosyltransferase (UGT) in Atlantic Wood killifish when compared to reference fish. In addition, a numerical rise in sulfotransferase (SULT) activity was noted, although overall level of activity measurable in killifish was low. Further, Cooper, Vogelbein, and Van Veld (1999) demonstrated that the xenobiotic transporter p-glycoprotein (Pgp, some of which are also known as ATP-binding cassette transporters or multidrug resistance proteins) was expressed at levels two- to threefold higher in Atlantic Wood fish than in reference killifish. Pgp, sometimes referred to as Phase III metabolizing enzymes, are membrane transport ATPases that are involved in the efflux of compounds. Although these proteins have been identified in a variety of normal tissues, they are also frequently elevated in multidrug-resistant cell lines and chemotherapy-resistant tumors (Abu-Qare, Elmasry, and Abou-Donia 2003). Thus far, the heritability of these elevated Phase II and III responses has not been investigated. Thus, it is not clear whether Pgp are components of heritable adaptation, acclimatory responses, or simply reflect toxic reactions to PAH exposure.

##### Oxidative stress

In addition to perturbations in AHR-mediated signaling, PAH produce toxicity via oxidative stress due to metabolism or photo-modification to products that generate reactive oxygen species (ROS) (Arfsten, Schaeffer, and Mulveny 1996). For this reason, Meyer et al. (2003a) hypothesized that upregulation of antioxidant defenses might be an important component of resistance to stressors in the Elizabeth River environment. Data demonstrated that exposure to Elizabeth River sediments led to increases in several antioxidant defenses, including total glutathione (GSH), glutathione reductase (GR) activity, and manganese superoxide dismutase (MnSOD) protein, in both Elizabeth River and reference fish. Studies with zebrafish established that, as with Elizabeth River sediments, a mixture of BNF and α-naphthoflavone (ANF) that produced cardiac malformations also induced expression of a battery of antioxidant response genes (Timme-Laragy et al. 2009). To further examine the role of oxidative stress in the synergistic toxicity of BNF and ANF, Timme-Laragy et al. (2009) used morpholino knockdown of NF-E2-related factor 2 (Nrf2) expression. Nrf2 is a transcription factor that controls expression of a variety of antioxidant defenses (Kaspar, Niture, and Jaiswal 2009), and knockdown of its transcription resulted in exacerbation of BNF and ANF mixture toxicity.

Given data showing the potential for PAH mixtures to generate oxidative stress, it was not surprising that Elizabeth River killifish differed from reference fish in their response to oxidative stressors (“pro-oxidants”). Elizabeth River F1 and F2 larvae were more resistant than reference larvae to the model pro-oxidant *t*-butyl hydroperoxide (Meyer et al. 2003a). Compared to reference fish, whole-body homogenates of Elizabeth River F1 or F2 larvae also displayed greater total oxygen scavenging capacity (TOSC), quantitatively elevated total GSH levels, and higher MnSOD protein expression. Total GSH levels were particularly increased in larval liver. In a subsequent study, wild-caught Elizabeth River adults exhibited higher hepatic total GSH and selenium-independent glutathione peroxidase (GPx) activity; however, selenium-dependent GPx activity was higher in reference fish (Bacanskas, Whitaker, and Di Giulio 2004). Mitochondrial lipid peroxidation was elevated in Elizabeth River fish relative to reference fish, despite the observed increases in antioxidant defenses. Overall, these studies suggest that Elizabeth River sediments and PAH mixtures generate oxidative stress and that killifish exhibit a variety of short-term physiological responses to this stress. Further, some antioxidant responses in Elizabeth River killifish appear to be heritable defenses that may contribute to overall resistant phenotype.

##### Downregulation of the aryl hydrocarbon receptor (AHR) pathway

The most pronounced and consistent biochemical alteration observed in the resistant killifish is marked recalcitrance to induction of Phase I metabolizing enzyme CYP1A (described previously). This conspicuous downregulation, along with the knowledge of the role CYP1 enzymes play in activation of PAH to more toxic and reactive metabolites, led the Di Giulio lab to pursue the hypothesis first proposed by Van Veld and Westbrook (1995) that suppression of CYP1A activity was a major component of resistance in Atlantic Wood killifish. To test this hypothesis and investigate the ramifications of exposure to PAH mixtures, reference population (King’s Creek) killifish embryos were exposed to AHR agonists while CYP1A activity was suppressed using chemical inhibitors (Wassenberg and Di Giulio 2004a; 2004b; Wassenberg et al. 2005). However, in contrast to reducing toxicity, combined exposure of embryos to an AHR agonist and a CYP inhibitor resulted in an unexpected and dramatic synergistic increase in cardiac teratogenesis. The combination of 11 μg/L of AHR agonist BNF with 100 μg/L of CYP1A inhibitor α-naphthoflavone (ANF) resulted in a near-maximal score on the deformity index used, despite the fact that neither compound elicited any observable teratogenicity on its own (Wassenberg et al. 2005). Comparable results were observed using a variety of AHR agonists (benzo[a]pyrene [BaP], BNF, and PCB-126) and CYP1A inhibitors (ANF, fluoranthene [FL], 2-aminoanthracene, piperonyl butoxide, carbazole, and dibenzothiophene) (Wassenberg and Di Giulio 2004a; Wassenberg et al. 2005). Similar marked enhancement of toxicity was noted when embryos were exposed to a mixture of diluted Elizabeth River pore water and ANF (Wassenberg and Di Giulio 2004b). To further investigate this phenomenon and confirm the role of CYP1A inhibition, targeted morpholino knockdown of CYP1A was performed. Our lab in collaboration with the Hahn laboratory (Woods Hole Oceanographic Institute) developed the morpholino gene knockdown technique for use in killifish and demonstrated that knockdown of CYP1A exacerbated BNF-induced toxicity in killifish (Matson et al. 2008). Collectively, these results have a number of important implications. Clearly, suppression of CYP1A alone is not only insufficient to provide the resistance observed in Elizabeth River killifish but in fact increases toxicity of AHR agonist-type PAH in naive fish. Further, at least with respect to embryotoxicity, data call into question the model of additive toxicity that is commonly assumed for PAH.

Thus far, the mechanisms by which CYP1A inhibition synergizes PAH toxicity remain to be determined. One hypothesis is that blockage of metabolism by CYP1A prevents degradation of PAH and prolongs its persistence in fish. This may serve to make PAH resemble DLC, perhaps explaining why cardiac teratogenesis generated by PAH mixtures closely resembles that induced by DLC. Another possibility is that inhibition of CYP1A shifts metabolism of the PAH to a different route that results in more toxic metabolites. In an attempt to address this hypothesis, Elizabeth River and reference embryos were exposed to a mixture of BaP and the CYP1A inhibitor PAH FL from 24 h post fertilization (hpf) to 120 hpf, at which point the presence of parent BaP and specific metabolites were measured (Wills et al. 2009). Coexposure to FL exerted minimal effects on the rate of BaP metabolism in either population; Elizabeth River embryos retained numerically more parent BaP. In addition, more of the only metabolite detected, BaP-9,10-dihydrodiol, was measured in Elizabeth River embryos. Because 9,10-dihydrodiol and its subsequent metabolite, 9,10-diol,7,8-epoxide, are less mutagenic than the alternatives (Stegeman and James 1985; Peltonen and Dipple 1995), Wills et al. (2009) postulated that this shift in metabolism might contribute to resistance in Elizabeth River killifish. Increased production of 9,10-dihydrodiol may be indicative of a rise in the ratio of epoxide hydrolase activity relative to CYP1A (Kleinow et al. 1998; Oesch 1988; Bauer et al. 1995; James and Little 1983). However, it should be noted that the Wills et al. (2009) study was hampered by detection limits for most BaP metabolites measured.

Given the results of the effect of blocking CYP1A on PAH toxicity, it appears likely that the refractory CYP1A response in Elizabeth River killifish might be part of a broader downregulation of the entire AHR pathway. The critical role of AHR in toxicity of both DLC and some PAH makes it a likely target for development of resistance. Other populations of fish, including killifish, were identified that are resistant to DLC (Wirgin and Waldman 2004), and several demonstrated resistance to both PAH and DLC. As indicated previously, Elizabeth River killifish are resistant to PCB-126 and to PAH (Meyer and Di Giulio 2002; Nacci, Champlin, and Jayaraman 2010). Killifish from New Bedford Harbor, MA are resistant to high levels of PCB at the site and also to PAH (Bello et al. 2001; Nacci et al. 1999; 2002) While tomcod (*Microgradus tomcod*) from the Hudson River, New York, are resistant to high levels of PCB contamination, their CYP response is refractory to induction by PCB but not by PAH (Roy et al. 2002). However, they are less susceptible to DNA damage by BaP than reference-site tomcod (Sorrentino et al. 2004). One hypothesis to explain development of resistance to different but related classes of contaminants in multiple populations and species is through modification of a single conserved gateway, such as the AHR.

Further evidence that PAH resistance in Elizabeth River killifish occurs through modification at the top of the AHR pathway was provided by analysis of transcriptional expression of hepatic CYP1A, AHR1, AHR2, ARNT2, and AHRR in adult fish treated with BNF (Meyer et al. 2003b). CYP1A, AHRR, and AHR2 expression were induced in reference fish, but not in Elizabeth River fish; AHR1 and ARNT2 were not differentially expressed. Data suggest suppression at the level of the AHR, since both CYP1A and AHRR are AHR regulated. It is also notable that there were no marked differences between populations in basal levels of expression of any genes examined, which implies that a difference in initial mRNA expression of AHR pathway genes does not confer resistance. Further, it is noteworthy that AHR2 was inducible in reference but not Elizabeth River fish. Although there is debate as to whether AHR is inducible by chemical treatment, one might expect that if it were, Elizabeth River fish direct from the field might display elevated basal levels due to PAH exposure in situ. This lack of induction of AHR2 in Elizabeth River fish, coupled with statistically indistinguishable basal levels of expression, may indicate that resistance is conferred through an insensitive or noninducible AHR2 in Elizabeth River fish. It is important to note, however, that expression of AHR2 was increased detectably in male fish from the Elizabeth River, indicating that these changes may have a sex-specific component. Thus far, these comparisons have only been made at the level of mRNA, not protein. Recent assessment in the Di Giulio lab of PAH-induced CYP mRNA expression in larvae and embryos provides further evidence that alterations occur prior to transcription of individual AHR-responsive genes. CYP1A, CYP1B1, and CYP1C1 were all induced by BaP exposure in larval reference but not Elizabeth River larvae (Wills et al. 2010b), and Elizabeth River embryos were also resistant to expression of the three CYP after exposure to multiple PAH and PCB-126 (Clark, Bone, and Di Giulio 2014). It is more likely that alterations at the level of AHR might be responsible for the observed changes in expression of various AHR pathway genes, rather than separate changes for each gene.

Morpholino knockdown studies provide evidence supporting the potential of modulation of AHR for conferring resistance to PAH and PAH mixtures. In zebrafish, morpholino knockdown of AHR2 was protective from synergistic cardiac toxicity induced by a mixture of BNF and ANF (Billiard et al. 2006) and by high doses of pyrene or benz[a]anthracene (Incardona et al. 2005; 2006). Further, work in the our lab showed that AHR2 knockdown in zebrafish protects from teratogenesis due to PAH mixtures, including BaP + FL, and benzo[k]fluoranthene (BkF) + FL (Van Tiem and Di Giulio 2011; Garner and Di Giulio 2012). Similarly Clark et al. (2010) demonstrated that knockdown of AHR2, but not AHR1, protected killifish from cardiac teratogenesis due to BNF, BkF, and PCB-126.

Mechanisms by which AHR mediates developmental toxicity of some PAH in fish, including killifish and zebrafish, are largely unknown. This phenomenon has received significantly more attention in the context of DLC, particularly TCDD. These studies suggested interactions between the AHR and other signaling genes and pathways involved with development, including Sox9b (Hofsteen et al. 2013), the COX2-thromboxane pathway (Teraoka et al. 2014), and the Wnt pathway (Schneider, Branam, and Peterson 2014). This remains an active area of research. It is likely that mechanisms revealed for DLC are relevant to PAH that are AHR agonists. A recent microarray study in our lab exploring PAH synergy, described earlier in this article, suggested a connection between the AHR pathway and perturbed calcium homeostasis affecting heart muscle contractility as a potential target (Jayasundara et al. 2015). Additional studies in this vein are underway.

However, there is also evidence that some of the components of PAH mixtures may produce cardiac toxicity independent of the AHR pathway. In particular, studies investigating the mechanisms underlying oil-mediated embryotoxicity found that some three- and four-ring PAH present in both oil and creosote mixtures (i.e., phenanthrene, dibenzothiophene, and pyrene) produced cardiac and circulatory abnormalities in zebrafish (Incardona, Collier, and Scholz 2004; 2005; 2006) . In one such study, morpholino knockdown of AHR2 blocked developmental cardiac toxicity induced by exposure to 5 μ*M* pyrene, but not that produced by exposure to 28 μ*M* phenanthrene (Incardona et al. 2005). Further, low-molecular-weight PAH are a major component of contamination in Elizabeth River sediments (Clark et al. 2013; Fang et al. 2014). Although the role of AHR-pathway-independent toxicity in the adaptation of Elizabeth River killifish has not been comprehensively examined, a recent effect-directed analysis of Elizabeth River pore water showed that in addition to toxicity induced by fractions containing high-molecular-weight PAH, a fraction dominated by low-molecular-weight PAH was also toxic to zebrafish embryos (Fang et al. 2014). A simplified mixture meant to simulate the relative concentrations of the dominant chemicals from that fraction (dibenzothiophene, phenanthrene, FL, pyrene, 1,2-benzofluorene, 1,2-benzanthracene, and chrysene) resulted in similar toxicity, but a mixture that matched the concentrations of the two most dominant compounds in the fraction (phenanthrene and FL) failed to produce an effect. In contrast, Brown et al. (2014) showed that exposure of zebrafish embryos to 6 μ*M* phenanthrene alone did not produce cardiotoxicity, but damage to the heart occurred when embryos were co-exposed to 6 μ*M* phenanthrene and 2.5 μ*M* FL.

### Fitness Trade-Offs and Cross-Resistance in PAH-Adapted Killifish

Although evolutionary theory predicts that rapid adaptation to single stressors is likely to be accompanied by concurrent fitness costs to populations, not all studies confirm that (Kinnison and Hairston 2007). Strong and rapid selection is thought to reduce overall population genetic variation and fitness following a population bottleneck. However, even relatively rapid development of resistance to chemical pollution may occur via a series of changes in populations, potentially avoiding such bottlenecks and their associated costs. Little evidence for the classical fitness costs of adaptation, that is, reduced fecundity, growth rate, and survival, has been identified in Elizabeth River killifish, but a broader interpretation might also include greater sensitivity to other stressors—chemical, physical and biological. Despite the significant PAH resistance exhibited by Elizabeth River killifish, they are not wholly adversely unaffected by contamination. As described previously, Elizabeth River killifish suffer from a variety of hepatic and extrahepatic neoplasms (Vogelbein et al. 1990; Fournie and Vogelbein 1994). These fish also seem to be more susceptible to diseases and have documented alterations in immune function (Meyer et al. 2005; Weeks, Warinner, and Mathews 1988; Faisal et al. 1991a; Meyer and Di Giulio 2003; Frederick, Van Veld, and Rice 2007; Kelly-Reay and Weeks-Perkins 1994). This may also be a direct effect of PAH exposure, as there is evidence that PAH exert a variety of effects on the immune system of fish (Reynaud and Deschaux 2006). Some of these effects are clearly an effect of long-term persistence in a heavily polluted environment, but others may be costs of adaptation.

To explore the consequences and fitness costs of adaptation, Meyer and Di Giulio (2003) compared responses of Elizabeth River and reference killifish to several stressors and to clean conditions. In clean conditions, survivorship of Elizabeth River F1 larvae was significantly lower than for reference larvae after 9 months. Interestingly, survivorship of F2 larvae was no different than for reference fish. A similar trend was observed for growth. In addition, reference larvae tolerated phototoxicity mediated by combined exposure to FL and ultraviolet (UV) light for longer than Elizabeth River larvae. However, Elizabeth River F1 larvae still survived longer when exposed to Elizabeth River sediments, regardless of UV exposure. In addition, Elizabeth River F1 and F2 larvae tolerated low O_2_ (0.5 mg/L) conditions for significantly less time than reference larvae in short-term acute exposures

Taking a nontargeted approach, Meyer et al. (2005) used suppressive subtractive hybridization to generate a differential display of genes expressed in livers of Elizabeth River killifish and a reference population. Some of the genes that were most strikingly decreased in the Elizabeth River fish were related to immune function, including Factor XI, a clotting factor also involved in complement activation, and complement components C3 and C9. Further, expression of UDP-glucose pyrophosphorylase was lower and expression of glucose 6-phosphatase was higher in Elizabeth River fish, suggesting impairment of aerobic energy metabolism and a shift away from energy production in mitochondria. This result is consistent with reduced tolerance of Elizabeth River killifish to hypoxia.

A series of studies by Jung and coworkers further explored the effect of PAH and of PAH adaptation on mitochondria of Elizabeth River and reference killifish. While the bulk of CYP1A protein and activity occurs in the endoplasmic reticulum of cells, it also is present in mitochondria. Jung and Di Giulio (2010) isolated killifish mitochondrial CYP1A and showed that it was induced by BaP in reference fish but not in Elizabeth River killifish. Using long-amplicon polymerase chain reaction (PCR), Jung and coworkers (2009a; 2009b) demonstrated that wild-caught Elizabeth River adults exhibited significantly greater levels of both mitochondrial DNA (mtDNA) and nuclear DNA (nDNA) damage in liver, brain, and muscle than reference fish.

Although wild-caught Elizabeth River killifish exhibited a higher basal level of DNA damage, they may actually be less susceptible to induction of DNA damage by PAH. Jung et al. (2009a) found that intraperitoneal (ip) injection of BaP resulted in a two- to sevenfold increase in DNA damage in reference fish, but less than twofold rise in Elizabeth River. This fits with the observations of Wills et al. (2009) indicating that Elizabeth River fish might be shifting metabolism of BaP away from the formation of DNA-damaging metabolites. Further, this correlates strongly with Wills et al. (2010a) showing that Elizabeth River killifish are less susceptible to BaP-induced liver toxicity and carcinogenesis. Data suggest that while Elizabeth River killifish display elevated rates of liver cancer due to living in an environment highly enriched in carcinogens, they are nonetheless more resistant to this PAH-mediated cancer than naive killifish. A reasonable hypothesis for this is that the downregulation of the AHR pathway in Elizabeth River killifish that confers resistance to acute toxicity of PAH also results in reduced activation of PAH to their ultimate mutagenic metabolites. However, it is conceivable that resistance to the acute effects, which readily parlay into effects on population dynamics, is the evolutionary driver for adaptations, not resistance to cancer, which is less tied to population dynamics.

In another investigation, Clark and Di Giulio (2012) determined whether PAH adaptation had trade-offs for Elizabeth River fish exposed to contaminants for which the mode of action (MOA) differed from that of PAH but that were known to interact with components of the AHR pathway. Larvae were challenged with neurotoxic insecticides, including those for which the adaptive suppression of the AHR pathway would be beneficial (chlorpyrifos; activated by CYP to the more toxic chlorpyrifos-oxon) and detrimental (permethrin; degraded by CYP to less toxic metabolites). Surprisingly, the Elizabeth River larvae were more resistant than reference larvae to both chlorpyrifos and permethrin, suggesting that other aspects of adaptation were contributing to resistance. Although some chemicals affect Elizabeth River fish equally, a chemical challenge to which Elizabeth River fish are more susceptible than reference fish, other than FL in combination with UV exposure, has not yet been tested (Bryan Clark, unpublished data).

**FIGURE 7.**
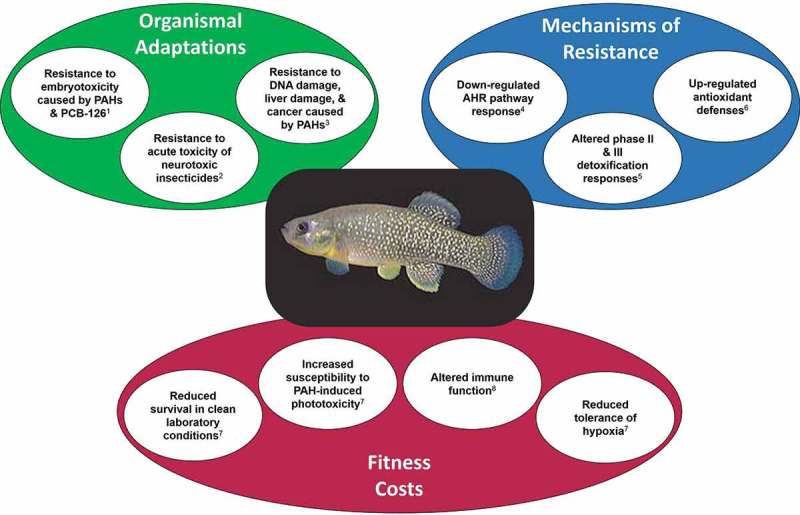
The “Elizabeth River phenotype,” summarizing organismal adaptations, underlying mechanisms, and fitness costs, based largely on studies of the Atlantic Wood industries population of Atlantic killifish. References: 1, Ownby et al. 2002, Meyer et al. 2002, Meyer and Di Giulio 2003, Wills et al. 2009, Nacci et al. 2010, Clark et al. 2014; 2, Clark et al. 2012; 3, Jung et al. 2009b, Wills et al. 2010b; 4, Van Veld and Westbrook 1995, Meyer et al. 2002, 2003b, Wills et al. 2010b, Nacci et al. 2010, Clark et al. 2013; 5, Van Veld et al. 1991, Armknecht et al. 1998, Cooper et al. 1999, Gaworecki et al. 2004; 6, Bacanskas et al. 2004, Meyer et al. 2003a; 7, Meyer and Di Giulio 2003; 8, Faisal et al. 1991, Kelly-Reay and Weeks-Perkins 1994, Frederick et al. 2007. Image of killifish used with permission from John Brill.

Key aspects of the “Elizabeth River phenotype” described in the forgoing sections are presented in Figure 7.

## POPULATION GENETICS AND ADAPTATION

Thus far, much of the work reviewed here focused on the nature and underlying mechanism of PAH tolerance in Elizabeth River killifish. However, the existence of the adapted Elizabeth River population along with contaminant-adapted killifish populations in New Bedford Harbor, MA, and Newark Bay, NJ, provides unique opportunities for investigating the effects of pollutants on selection and genome-level responses to environmental contaminants.

There are several explanations for the persistence of a killifish population in the highly contaminated Atlantic Wood Superfund site. One is that they have developed heritable adaptations that enable their survival; much of the work described previously supports that conclusion. Alternately, the Atlantic Wood subpopulation may suffer a high rate of mortality, but continually replenish via immigration from nearby subpopulations. Various investigations used allozyme frequency (Mulvey et al. 2002) and mtDNA haplotype diversity (Mulvey et al. 2003) to test these possibilities. In these studies genetic diversity was compared among subpopulations throughout the Elizabeth River estuary and reference populations in the nearby York River. The Elizabeth River subpopulations were located in sites that varied in degree of sediment PAH contamination. In both studies, data demonstrated that fish from the Atlantic Wood site were genetically distinct from the other subpopulations investigated. Fish from a second, moderately contaminated Elizabeth River site were less distinct from other subpopulations. Overall, genetic distance between subpopulations was not correlated with geographic distance but rather with degree of PAH contamination at the site of collection. Thus, immigration from subpopulations does not appear to be a significant mechanism for supporting continued existence of the AW population. There is evidence, however, indicating that such immigration might occur with DLC-adapted populations (Roark et al. 2005; McMillan et al. 2006).

Recently, Clark et al. (2013) compared the level of resistance to a variety of aryl hydrocarbons among Elizabeth River killifish subpopulations from throughout the estuary, most of which were collected in or near the sites used by Mulvey and coworkers (2002; 2003). Unlike the Atlantic Wood population, some of the subpopulations exhibited a high degree of AHR pathway activation, as measured by EROD activity, but were also resistant to aryl hydrocarbon-induced teratogenesis. Further, various subpopulations exhibited unique patterns of response to the differing hydrocarbons tested. The varied patterns of resistance to teratogenesis and AHR pathway activation suggest that PAH adaptation in the Elizabeth River killifish meta-population is multigenic, with highly resistant Atlantic Wood fish exhibiting all aspects of resistance and various subpopulations only sharing some of the resistance mechanisms.

Further, the degree of aryl hydrocarbon resistance was not highly correlated with the level of PAH at the subpopulation collection site. These unique patterns of subpopulation response and lack of correlation to contaminant level are also notable given the previous observations of strong correlations between PAH level and genetic distance (Mulvey et al. 2002; 2003) and resistance (Ownby et al. 2002). Mulvey et al. (2002; 2003) estimated a rate of migration sufficient to maintain significant gene flow among subpopulations; the modeled effective migration rates were 9.6 migrants per generation for juveniles and 17.5 migrants per generation for adults. It was hypothesized that the Atlantic Wood population remains genetically distinct despite the migration rate because immigrants are unable to survive in the harsh conditions. However, it is possible that emigrants from the Atlantic Wood site might be driving the appearance of adaptation at sites with low levels of PAH contamination that was observed. Moreover, the transmission and fixation of only some components of a multigenic adaptation to other subpopulations might explain the unique patterns and incomplete resistance displayed by the individual subpopulations. High migration rates may also serve to help maintain overall genetic diversity, given that strong selection for a particular adaptive parameter might lower overall genetic diversity. Conversely, pollutant stress may reduce genetic variability in populations by reducing population size through loss of sensitive individuals and reduced fecundity. However, Mulvey et al. (2002); 2003) noted no marked loss of genetic diversity in either study of Elizabeth River killifish.

In studies comparing aryl hydrocarbon-adapted killifish populations from the Atlantic Wood Industries Superfund site and Superfund sites in New Bedford Harbor, MA, and Newark Bay, NJ, Oleksiak and coworkers searched for transcriptomic loci linked to contaminant exposure and adaptation. Hypothesizing that pollutant exposure would necessitate changes in metabolic gene expression to provide energy for protection against toxicity, a metabolic cDNA array was utilized to compare gene expression in brain (Fisher and Oleksiak 2007) and liver (Oleksiak 2008) between each pollution-adapted population and two nearby matched reference populations. Compared to its reference sites, 5% of the examined genes (13 of 260) were differentially expressed in brains of Elizabeth River killifish, but this was reduced to only one gene after Bonferroni correction. In addition, only two genes were shared among all three Superfund populations (NADH-ubiquinone oxidoreductase AGG subunit precursor, a subunit in oxidative phosphorylation complex I, and thioredoxin, an oxidoreductase that facilitates the reduction of other proteins). In livers of Elizabeth River killifish, 8% of examined genes (20 of 250) were differentially expressed compared to reference populations; this reduced to 2 of 250 after Bonferroni correction. Again, a small number of genes were identified that were differentially expressed in all three aryl hydrocarbon-adapted populations (acyl-coenzyme A [CoA]-binding protein, the MNLL subunit of NADH-ubiquinone oxidoreductase, and thioredoxin). However, none of these genes exhibited consistent expression patterns among the polluted populations. Evidence indicated that the relative lack of overlap among the gene expression patterns of the three aryl hydrocarbon-adapted populations reflects different routes of adaptation and different pollutant exposure. However, previous studies found a great deal of overlap in the adaptive responses of the three populations (Wirgin and Waldman 2004); thus, it is also possible that the limited number of genes or the sensitivity of the array was unable to fully capture the adaptive changes. In contrast, Whitehead et al. (2012; 2010) observed convergent transcriptomic responses in comparisons of PCB-126 embryotoxicity exhibited by several DLC-adapted populations (the Elizabeth River population was not tested). In these studies, embryos from tolerant and sensitive populations were exposed to PCB-126 and at 10 d postfertilization were assessed for both phenotypic and transcriptomic responses. Among the major transcriptomic responses in the sensitive populations were a decrease in expression of cardiovascular-system-related genes (Whitehead et al. 2010) and an increase in expression of genes associated with the AHR pathway (Whitehead et al. 2010; 2012); these responses were absent in the adapted populations. Finally, similar to Mulvey et al. (2002; 2003), Oleksiak et al. (2002; 2005) also did not observe any reduction in variance in gene expression due to contaminant adaptation.

Subsequently, Williams and Oleksiak (2008) compared allele frequencies of amplified fragment length polymorphisms (AFLP) among the same pollutant-adapted and paired reference populations used in the gene expression studies. The goal was to identify genomic loci with large differences in allele frequency between the aryl hydrocarbon-adapted and reference populations, but that were not also highly different between the pair of reference populations; these outlier loci might be involved in adaptation and pollutant responses. Three percent (9 of 299) of the loci scored were identified as outliers in comparison of the Elizabeth River killifish to two reference populations. Of these, six were found in individual comparisons of the Elizabeth River population to each reference population that were not found in comparison of reference populations to each other. The Elizabeth River population shared two outlier loci with the New Bedford Harbor fish and two with the Newark Bay fish. No outliers were shared among all three adapted populations; rather, most of the identified loci were unique to each adapted population. This may be attributed to linked loci dragging different polymorphisms to fixation in each population, even if the locus driving selection was actually the same in each population, or the adaptation might differ among the populations. However, as indicated previously, populations are faced with similar contaminant profiles and exhibit many similar adaptive responses, so it is surprising that more shared loci were not identified. Williams and Oleksiak (2008) also postulated that other selective pressures, such as predation or food availability, may drive differences among populations. It is interesting to consider that these differences may also be secondary effects arising from differing effects of the contamination in each ecosystem.

Because of the multiple lines of evidence pointing to the AHR pathway as a target for adaptation, Proestou et al. (2014) compared the pattern of genetic variation in single nucleotide polymorphisms (SNP) in 42 AHR pathway genes in 4 DLC-tolerant killifish populations and nearby reference sites. Pairwise comparisons of geographically close tolerant and sensitive populations uncovered differences in allelic composition at AHR1 and 2, cathepsin Z, CYP1A and CYP3A30, and the NADH dehydrogenase subunits, but variation in AHR2 and CYP1A was strongest across all of the tolerant versus sensitive population pairings, supporting a role for alterations of the AHR pathway in the adaptive response of multiple populations. Further, Proestou et al. (2014) did not find any marked reduction in overall genetic variation in the pollutant-adapted populations, in agreement with multiple previous studies.

## CONCLUDING REMARKS

This review is by no means the first discovery concerning pollution driving animal evolution. The phenomenon of industrial melanism in populations of the peppered moth (*Biston betularia*) in England and Wales is perhaps the iconic example of pollution (in this case, soot emitted by coal burning) affecting evolution (Cook 2003). In this case, natural selection favored the dark, melanic phenotype (carbonaria) over the normally lighter, “peppered” phenotype (typica), apparently due to reduced avian predation due to the camouflage afforded against the dark, soot-covered tree trunks the moths utilized. Following control of emissions, the carbonaria phenotype gradually declined in relative abundance (Cook 2003). There are numerous reports of insects evolving resistance to insecticides, including mechanisms associated with different classes of insecticides. In recent years, this has garnered particular attention in relation to mosquito control, in part due to the key role these insects play in malaria transmission (Lenormand et al. 1999; Wondji et al. 2008). As alluded to earlier, there are other examples of pollution tolerance in fish populations, some of which appear to be inheritable and thus of evolutionary significance. These include DLC-resistant killifish populations in New Jersey, Massachusetts, and Connecticut (Prince and Cooper 1995; Nacci, Champlin, and Jayaraman 2010), resistance to DLC and PAH in Gulf killifish (*Fundulus grandis*) in Texas (Oziolor et al. 2014), resistance to methylmercury, also in New Jersey killifish (Weis et al. 1981), and resistance to cadmium in lab-reared least killifish (*Heterandria formosa*) (Xie and Klerks 2003). The subject of chemical toxicity resistance in fish was reviewed by Van Veld and Nacci (2008); however as they point out, most examples of this phenomenon did not include studies to determine whether observed adaptations were genetically based.

The Elizabeth River story of pollution-driven evolution in killifish is compelling for several reasons. The degree of resistance exhibited by the Atlantic Wood Industries population is striking and has garnered attention of numerous investigators over the years; thus, there is a wealth of information concerning this population gathered from a number of perspectives, including toxicology, ecology, genomics, physiology, risk assessment and management. Other subpopulations in the river have also been identified that reveal varying degrees of adaptation, and that, together with the Atlantic Wood population, provide fertile grounds for studying pollution-driven evolution, underlying mechanisms, fitness costs, and impacts of remediation. Regarding remediation, two of the former creosote sites (Atlantic Wood Industries and Eppinger and Russell) are undergoing active remediation, and remediation of the third, Republic Creosoting, is planned. Thus, these sites, together with other sites in the river not associated with wood treatment, provide excellent opportunities for examining how specific subpopulations change during and post remediation. Also noteworthy is the chemical class at the center of this story—PAH. While the major source of PAH in the Elizabeth River is creosote that apparently is no longer used for wood treatment in the area, PAH are ubiquitous environmental contaminants that appear to be gradually increasing due to the use of fossil fuels and concomitant population growth and urbanization (Van Metre and Mahler 2005). In addition, these compounds are the chemicals of greatest concern following oil spills such as the 2010 Deepwater Horizon explosion in the Gulf of Mexico, the impacts of which included effects on the Gulf killifish (Dubansky et al. 2013). Thus, the gradient of PAH contamination present at specific sites in the Elizabeth River provides a powerful platform on which to understand organismal and population-level effects of these chemicals in a real-world ecosystem.

Work to date in this system has yielded several significant findings, and raised important questions. Demonstrating that the resistance was genetically based and thus evolutionarily meaningful is important; perhaps not surprisingly, there are relatively few clear examples of pollution driving evolution in free-living vertebrates. Interestingly, several of the other demonstrations of this also were observed in the Atlantic killifish, supporting the utility of this species as sentinel for Atlantic coast estuaries; the closely related Gulf killifish has a similar utility on that coast. Considerable insight has been gained concerning mechanisms underlying the resistance, which in turn provides a unique opportunity to address mechanisms of PAH-mediated toxicity. Among mechanisms observed, downregulation of the AHR in the Atlantic Wood populations has been the most pronounced and consistent signal. This is similar to observations of DLC-adapted killifish in the northeastern United States. Mechanisms that underlie this downregulation and those by which the AHR mediates toxicity in nonadapted killifish (and other animals) remain important subjects of inquiry.

The observation that the most sensitive endpoint to exposures to extracts from the Atlantic Wood site and to simple PAH mixtures in nonadapted killifish (and also to zebrafish) was embryonic development of the cardiovascular system was a significant finding; this effect is postulated to be the evolutionary driver of resistance in the Atlantic Wood population. At the time of first observing this (approximately 2000), this effect was somewhat surprising, as it was not a “textbook” effect of PAH, as opposed, for example, to cancer (upon which human risks for PAH are based). The only previous example of a PAH perturbing cardiovascular development we are aware of is that of retene (7-isopropyl-1-methylphenanthrene, associated with pulp and paper mills) in zebrafish and rainbow trout (Billiard, Querbach, and Hodson 1999); retene also induced CYP1A activity (Brinkworth et al, 2003), suggesting that it is an agonist for the AHR.

A related and striking finding emanating from the Elizabeth River killifish studies was the unanticipated finding of the potent synergy between PAH that are AHR agonists (e.g., BaP) and those that are inhibitors of CYP1A (e.g., FL); these two representatives are among the most prevalent PAH at the Atlantic Wood site. This synergy has important ramifications for PAH risk assessments. Risk assessments for classes of chemicals often assume additivity; that is, the toxicity of a mixture of a given class may be predicted based on adding the relative toxic potencies and concentrations in the mixture, with potencies of component chemicals based on a well-characterized representative of high potency. This is referred to as the toxic equivalency approach and has been widely used in risk assessments of DLC in which TCDD serves as the reference compound (Van Den Berg et al. 2006). Risk assessments for PAH do not appear as formalized at present, but approaches implicitly assume additivity, which is explicitly assumed for DLC. For human risk assessments of PAH, cancer is the key endpoint and BaP is often the reference compound (U.S. EPA 2010). For ecological risks there appears to be no reference compound but the toxicities of individual PAH generally based on acute toxicities are added after adjusting for relative concentrations in the mixture of concern (Wu et al. 2011). Studies based on Atlantic Wood sediments suggest that this approach likely greatly underestimates risks posed by some PAH mixtures, particularly those with relatively high proportions of higher molecular weight AHR agonists—typical of pyrogenic PAH mixtures. This may be less true for petrogenic mixtures in which lower molecular weight PAHs appear to dominate toxicity, as in the case of oil spills (Incardona, Collier, and Scholz 2004).

The phenomenon of genetic adaptations to pollution that decreases sensitivity to exposures has other implications for risk assessment and environmental management. An argument could be made that free-living organisms with relatively short generation times may evolve to thrive in the face of chronic pollution that is of a consistent nature (as is the case for the Elizabeth River). However, evolutionary theory posits that genetic adaptations to a potent driver of natural selection will reduce genetic diversity and incur fitness costs in the adapted population (Ribeiro and Lopes 2013). While it is unclear whether pollution in the river reduced genetic diversity, genetic structure was altered in several subpopulations, and, importantly, fitness costs were incurred in the only subpopulation that was examined intensively in this regard to date (Atlantic Wood). This subject merits additional scrutiny and is an active area of investigation currently; preliminary results suggest fitness costs in other subpopulations of Elizabeth River killifish, with degree of costs roughly tracking degree of pollution resistance. In any event, to base risk assessments or management decisions on the observation that organisms evolve to survive pollution seems inappropriate. Other important considerations are the likely complex ramifications that evolved adaptations in the killifish have for other members of the community and the ecosystem overall. For example, one would predict that the adapted killifish accumulate greater amounts of PAH due to reduced metabolism, thereby increasing exposure to their predators.

A question that arises from this review is that of potential implications for human health. Given the long generation time of humans versus killifish, the relevance of the evolutionary component appears limited. However, the observed effects of PAH, including synergistic mixtures, on vertebrate development may be relevant in consideration of the ubiquity of this class of chemicals and their importance as air pollutants (Boström et al. 2002). However, there are important differences between fish embryos and human fetuses as targets for PAH. For fish embryos, PAH exposures are likely to occur either directly from the water column, or via vitellogenin, the yolk protein synthesized in the maternal liver that was shown to transfer BaP to killifish eggs (Monteverdi and Di Giulio 2000). Human fetal exposures would likely occur from maternal blood flow via the placenta. Thus, in the case of humans, there would be greater opportunity for PAH to be metabolized before they reached the fetus than in the case of fish embryos. Moreover, there are no apparent reports of PAH exposures to fetuses (carried by mothers that are smokers, for example) having effects on the developing cardiovascular system. This would be a more difficult consequence to observe in humans versus fish. However, studies by Columbia University investigators following a cohort of children born to nonsmoking mothers in New York City revealed strong associations between cord blood concentrations of PAH during pregnancy and subsequent adverse intellectual and behavioral outcomes in young children (Perera et al. 2012). These studies suggest an effect of PAH on human neurodevelopment. Current studies at Duke University are investigating behavioral effects in killifish and zebrafish at exposures below those producing frank cardiovascular effects. Thus, these human and fish studies appear to have relevance for one another.

**FIGURE 8.**
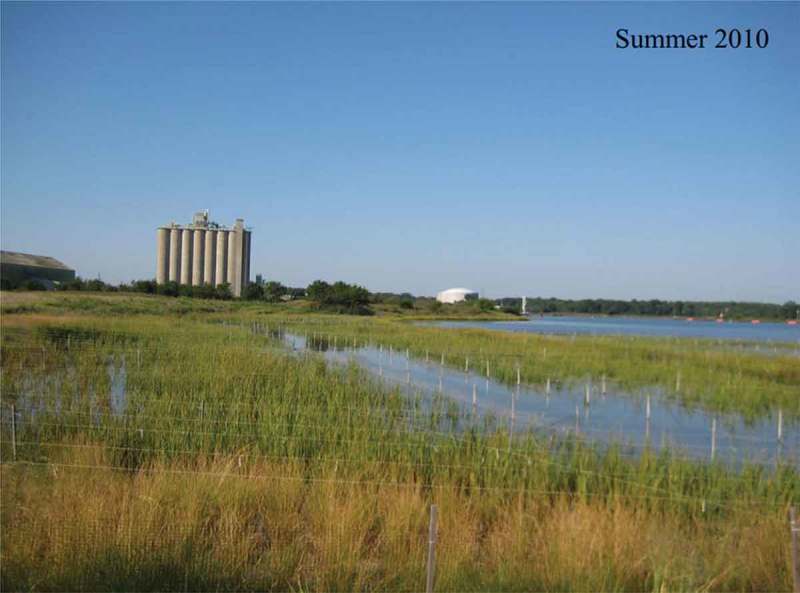
Wetlands restoration at Money Point, where Eppinger and Russell facilities operated previously. Credit—Elizabeth River Project.

**FIGURE 9.**
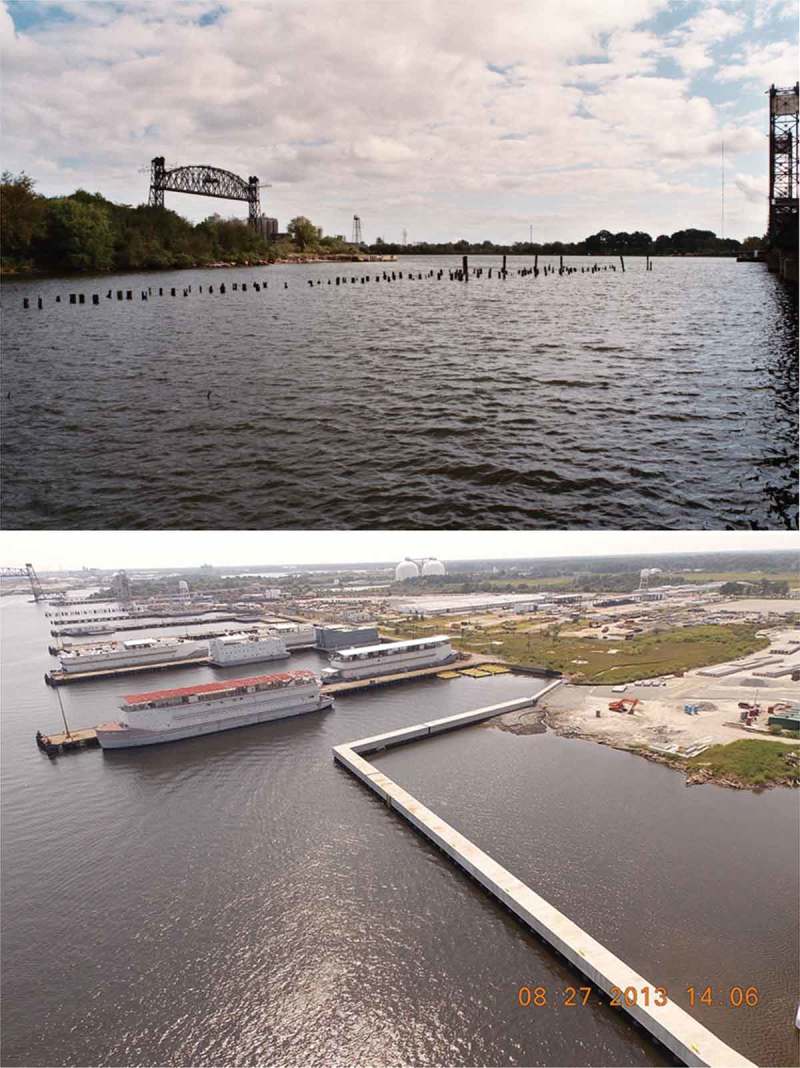
Photographs of the Atlantic Wood Industries site before (top, 2007; credit—Bryan Clark) and after remediation including the construction of a retaining wall in 2014 (credit—Elizabeth River Project).

Finally, while this story revolves around the degradation of an estuarine ecosystem due to human activities, it is important to conclude with the good news of ongoing efforts to restore the Elizabeth River. Coordinated efforts including, among others, the U.S. EPA, U.S. Army Corps of Engineers, National Oceanic and Atmospheric Administration (NOAA), the Virginia Department of Environmental Quality, and area towns (Chesapeake, Norfolk, Portsmouth, and Virginia Beach) and industries, are making significant progress in removing and limiting inputs of pollutants into the system, restoring habitats including wetlands, and increasing public awareness of the value of a restored Elizabeth River. Many of these efforts are being coordinated by the Elizabeth River Project, a non-profit organization incorporated in 1993 (website: http://www.elizabethriver.org). The project’s goal is to make the Elizabeth River swimmable and fishable by the year 2020; it also is involved with projects on the Lafayette River. Among the notable projects overseen by the Elizabeth River Project is the revitalization of Money Point, an area that includes the former creosoting facility operated by Eppinger and Russell (Figure 2). This ambitious project includes removal of hotspots of PAH contaminated sediments, prevention of future upland inputs of contaminants, enhancing the co-existence of industrial, community and ecological health, and the restoration of wetlands and other wildlife habitats. This project is well underway; contaminated sediments have been removed from the Eppinger and Russell site (with the assistance of the current landowner, Hess Corporation) and wetland restoration there is in progress (Figure 8). Also, a retaining wall surrounding the most contaminated sediments adjoining the former Atlantic Wood Industries Superfund site was recently completed (summer, 2014; Figure 9), and related restoration activities are planned.

Thus, there is good reason for optimism for the health of the Elizabeth River, an attitude not present several decades ago when a spokesman for the Virginia State Water Control Board stated, “It is questionable whether or not this body of water can be restored” (Yarsinke 2007, 330). Among other approaches, the Atlantic killifish provides a powerful sentinel for gauging the success of revitalization efforts in this historic American river.

## FUNDING

Portions of the research described in this review were supported by the National Institute of Environmental Health Sciences (NIEHS)-supported Duke University Superfund Research Center (P42ES010356) and Duke Integrated Toxicology and Environmental Health Program (T32ES07031).

## References

[CIT0001] Able K. W., Hagan S. M., Kovitvongsa K., Brown S. A., Lamonaca J. C. (2007). Piscivory by the mummichog (*Fundulus heteroclitus*): Evidence from the laboratory and salt marshes. *Journal of Experimental Marine Biology and Ecology*.

[CIT0002] Abraham B. J. (1985). Species profiles, life histories and environmental requirements of coastal fishes and invertebrates of mid-Atlantic USA: Mummichog and striped killifish. *U.S. Fish and Wildlife Service Biological Report*.

[CIT0003] Abu-Qare A. W., Elmasry E., Abou-Donia M. B. (2003). A role for P-glycoprotein in environmental toxicology. *Journal of Toxicology and Environmental Health, Part B*.

[CIT0004] Allen E. A., Fell P. E., Peck M. A., Gieg J. A., Guthke C. R., Newkirk M. D. (1994). Gut contents of common mummichogs, *Fundulus heteroclitus* L., in a restored impounded marsh and in natural reference marshes. *Estuaries*.

[CIT0005] Arfsten D. P., Schaeffer D. J., Mulveny D. C. (1996). The effects of near ultraviolet radiation on the toxic effects of polycyclic aromatic hydrocarbons in animals and plants: A review. *Ecotoxicology and Environmental Safety*.

[CIT0006] Armknecht S. L., Kaattari S. L., Van Veld P. A. (1998). An elevated glutathione *S*-transferase in creosote-resistant mummichog (*Fundulus heteroclitus*). *Aquatic Toxicology*.

[CIT0007] Armstrong P. B., Child J. S. (1965). Stages in the normal development of *Fundulus heteroclitus*. *Biological Bulletin*.

[CIT0008] Bacanskas L. R., Whitaker J., Di Giulio R. T. (2004). Oxidative stress in two populations of killifish (*Fundulus heteroclitus*) with differing contaminant exposure histories. *Marine Environmental Research*.

[CIT0009] Bauer E., Guo Z. Y., Ueng Y. F., Bell L. C., Zeldin D., Guengerich F. P. (1995). Oxidation of benzo[a]pyrene by recombinant human cytochrome P450 enzymes. *Chemical Research in Toxicology*.

[CIT0010] Bello S. M., Franks D. G., Stegeman J. J., Hahn M. E. (2001). Acquired resistance to Ah receptor agonists in a population of Atlantic killifish (*Fundulus heteroclitus*) inhabiting a marine Superfund site: In vivo and in vitro studies on the inducibility of xenobiotic metabolizing enzymes. *Toxicological Sciences*.

[CIT0011] Bernardi G., Sordino P., Powers D. A. (1993). Concordant mitochondrial and nuclear-DNA phylogenies for populations of the teleost fish *Fundulus heteroclitus*. *Proceedings of the National Academy of Sciences of the United States of America*.

[CIT0012] Bieri R. H., Hein C., Huggett R. J., Shou P., Slone H., Smith C., Su C. W. (1986). Polycyclic aromatic hydrocarbons in surface sedicments from the Elizabeth River subesturary. *International Journal of Environmental Analytical Chemistry*.

[CIT0013] Billiard S. M., Meyer J. N., Wassenberg D. M., Hodson P. V., Di Giulio R. T. (2008). Nonadditive effects of PAHs on early vertebrate development: Mechanisms and implications for risk assessment. *Toxicological Sciences*.

[CIT0014] Billiard S. M., Querbach K., Hodson P. V. (1999). Toxicity of retene to early life stages of two freshwater fish species. *Environmental Toxicology and Chemistry/SETAC*.

[CIT0015] Billiard S. M., Timme-Laragy A. R., Wassenberg D. M., Cockman C., Di Giulio R. T. (2006). The role of the aryl hydrocarbon receptor pathway in mediating synergistic developmental toxicity of polycyclic aromatic hydrocarbons to zebrafish. *Toxicological Sciences*.

[CIT0016] Boström C.-E., Gerde P., Hanberg A., Jernström B., Johansson C., Kyrklund T., Rannug A., Törnqvist M., Victorin K., Westerholm R. (2002). Cancer risk assessment, indicators, and guidelines for polycyclic aromatic hydrocarbons in the ambient air. *Environmental Health Perspectives*.

[CIT0017] Brinkworth L. C., Hodson P. V., Tabash S., Lee P. (2003). Cyp1a induction and blue sac disease in early developmental stages of rainbow trout (*Oncorhynchus mykiss*) exposed to retene. *Journal of Toxicology and Environmental Health A*.

[CIT0018] Brown D. R., Clark B. W., Garner L. V. T., Di Giulio R. T. (2014). Zebrafish cardiotoxicity: The effects of CYP1A inhibition and AHR2 knockdown following exposure to weak aryl hydrocarbon receptor agonists. *Environment Sciences Pollution Researcher*.

[CIT0019] Burnett K. G., Bain L. J., Baldwin W. S., Callard G. V., Cohen S., Di Giulio R. T., Evans D. H., Gomez-Chiarri M., Hahn M. E., Hoover C. A., Karchner S. I., Katoh F., MacLatchy D. L., Marshall W. S., Meyer J. N., Nacci D. E., Oleksiak M. F., Rees B. B., Singer T. D., Stegeman J. J., Towle D. W., Van Veld P. A., Vogelbein W. K., Whitehead A., Winn R. N., Crawford D. L. (2007). *Fundulus* as the premier teleost model in environmental biology: Opportunities for new insights using genomics. *Comparative Biochemical Physiological D-Genom Proteom*.

[CIT0020] Chu F. L. E., Hale R. C. (1994). Relationship between pollution and susceptibility to infectious disease in the eastern oyster, *Crassostrea virginica*. *Marine Environmental Research*.

[CIT0021] Chu F. L. E., Volety A. K., Hale R. C., Huang Y. Q. (2002). Cellular responses and disease expression in oysters (*Crassostrea virginica*) exposed to suspended field-contaminated sediments. *Marine Environmental Research*.

[CIT0022] Clark B. W., Bone A. J., Di Giulio R. T. (2014). Resistance to teratogenesis by F1 and F2 embryos of PAH-adapted *Fundulus heteroclitus* is strongly inherited despite reduced recalcitrance of the AHR pathway. *Environment Sciences Pollution Researcher*.

[CIT0023] Clark B. W., Cooper E. M., Stapleton H. M., Di Giulio R. T. (2013). Compound- and mixture-specific differences in resistance to polycyclic aromatic hydrocarbons and PCB-126 among *Fundulus heteroclitus* subpopulations throughout the Elizabeth River Estuary (Virginia, USA). *Environmental Science & Technology*.

[CIT0024] Clark B. W., Di Giulio R. T. (2012). *Fundulus heteroclitus* adapted to PAHs are cross-resistant to multiple insecticides. *Ecotoxicology*.

[CIT0025] Clark B. W., Matson C. W., Jung D., Di Giulio R. T. (2010). AHR2 mediates cardiac teratogenesis of polycyclic aromatic hydrocarbons and PCB-126 in Atlantic killifish (*Fundulus heteroclitus*). *Aquatic Toxicology*.

[CIT0026] Cochran R. E., Burnett L. E. (1996). Respiratory responses of the salt marsh animals, *Fundulus heteroclitus, Leiostomus xanthurus*, and *Palaemonetes pugio* to environmental hypoxia and hypercapnia and to the organophosphate pesticide, azinphosmethyl. *Journal of Experimental Marine Biology and Ecology*.

[CIT0027] Cook L. M. (2003). The rise and fall of the Carbonaria form of the peppered moth. *Quarterly Review of Biology*.

[CIT0028] Cooper P. S., Vogelbein W. K., Van Veld P. A. (1999). Altered expression of the xenobiotic transporter P-glycoprotein in liver and liver tumours of mummichog *Fundulus heteroclitus* from a creosote-contaminated environment. *Biomarkers*.

[CIT0029] Crawford D. L., Oleksiak M. F. (2007). The biological importance of measuring individual variation. *Journal of Experimental Biology*.

[CIT0030] Crawford D. L., Powers D. A. (1989). Molecular basis of evolutionary adaptation at the lactate dehydrogenase-B locus in the fish *Fundulus heteroclitus*.. *Proceedings of the National Academy of Sciences of the United States of America*.

[CIT0031] Crawford D. L., Powers D. A. (1992). Evolutionary adaptation to different thermal environments via transcriptional regulation. *Molecular Biology and Evolution*.

[CIT0032] Degnan K. J., Karnaky K. J., Zadunaisky J. A. (1977). Active chloride transport in the in vitro opercular skin of a teleost (*Fundulus heteroclitus*), a gill-like epithelium rich in chloride cells. *Journal of Physiology*.

[CIT0033] DiMichele L., Powers D. A. (1982). Physiological basis for swimming endurance differences between ldh-B genotypes of *Fundulus heteroclitus*. *Science*.

[CIT0034] Dubansky B., Whitehead A., Miller J. T., Rice C. D., Galvez F. (2013). Multitissue molecular, genomic, and developmental effects of the Deepwater Horizon oil spill on resident Gulf killifish (*Fundulus grandis*). *Environmental Science & Technology*.

[CIT0035] Dunson W. A., Fricano P., Sadinski W. J. (1993). Variation in tolerance to abiotic stresses among sympatric salt-marsh fish. *Wetlands*.

[CIT0036] Eisler R. (1986). Use of *Fundulus heteroclitus* in pollution studies. *American Zoologist*.

[CIT0037] Evans D. H. (2008). Teleost fish osmoregulation: What have we learned since August Krogh, Homer Smith, and Ancel Keys. *AJP: Regulatory, Integrative and Comparative Physiology*.

[CIT0038] Evans D. H., Piermarini P. M., Choe K. P. (2005). The multifunctional fish gill: Dominant site of gas exchange, osmoregulation, acid-base regulation, and excretion of nitrogenous waste. *Physiological Reviews*.

[CIT0039] Faisal M., Weeks B. A., Vogelbein W. K., Huggett R. J. (1991). Evidence of aberration of the natural cytotoxic cell activity in *Fundulus heteroclitus* (Pisces: Cyprinodontidae) from the Elizabeth River, Virginia. *Veterinary Immunology and Immunopathology*.

[CIT0040] Fang M. L., Getzinger G. J., Cooper E. M., Clark B. W., Garner L. V. T., Di Giulio R. T., Ferguson P. L., Stapleton H. M. (2014). Effect-directed analysis of Elizabeth River Porewater: Developmental toxicity in zebrafish (*Danio rerio*). *EnvironToxicol Chemical*.

[CIT0041] Fisher M. A., Oleksiak M. F. (2007). Convergence and divergence in gene expression among natural populations exposed to pollution. *BMC Genomics*.

[CIT0042] Foreso C., Taylor E., Wanser W., Nesbitt E. (1985). The position and competitiveness of the United States in world coal trade: Report on investigation no. 332-182 under section 332(b) of the Tariff Act of 1930.

[CIT0043] Fournie J. W., Vogelbein W. K. (1994). Exocrine pancreatic neoplasms in the mummichog (*Fundulus heteroclitus*) from a creosote-contaminated site. *Toxicologic Pathology*.

[CIT0044] Frederick L. A., Van Veld P. A., Rice C. D. (2007). Bioindicators of immune function in creosote-adapted estuarine killifish, *Fundulus heteroclitus*. *Journal of Toxicology and Environmental Health A*.

[CIT0045] Garner L. V. T., Di Giulio R. T. (2012). Glutathione transferase pi class 2 (GSTp2) protects against the cardiac deformities caused by exposure to PAHs but not PCB-126 in zebrafish embryos. *Comparative Biochemistry and Physiology Part C: Toxicology & Pharmacology*.

[CIT0046] Gaworecki K. M., Rice C. D., Van Den Hurk P. (2004). Induction of phenol-type sulfotransferase and glucuronosyltransferase in channel catfish and mummichog. *Marine Environmental Research*.

[CIT0047] Gonzalez H. O., Roling J. A., Baldwin W. S., Bain L. J. (2006). Physiological changes and differential gene expression in mummichogs (*Fundulus heteroclitus*) exposed to arsenic. *Aquatic Toxicology*.

[CIT0048] Gonzalez R. J., Mason C. H., Dunson W. A. (1989). Anomalous tolerance to low pH in the estuarine killifish *Fundulus heteroclitus*. *Comparative Biochemistry and Physiology Part C: Comparative Pharmacology*.

[CIT0049] Greaney G. S., Place A. R., Cashon R. E., Smith G., Powers D. A. (1980). Time coruse of changes in enzyme activites and blood respiratory properties of killifish during long-term acclimation to hypoxia. *Physiological Zoology*.

[CIT0050] Greaney G. S., Powers D. A. (1977). Cellular regulation of an allosteric modifier of fish haemoglobin. *Nature*.

[CIT0051] Griffith R. W. (1974). Environment and salinity tolerance in the genus Fundulus. *Copeia*.

[CIT0052] Hargis W. J., Roberts M. H., Zwerner D. E. (1984). Effects of contaminated sediments and sediment-exposed effluent water on an estuarine fish: Acute toxicity. *Marine Environmental Research*.

[CIT0053] Hargis W. J., Jr. D. E., Zwerner D. A., Thoney K. L., Kelly, Warinner J. E. (1989). Neoplasms in mummichogs from the Elizabeth River, Virginia. *Journal of Aquatic Animal Health*.

[CIT0054] Hofsteen P., Plavicki J., Johnson S. D., Peterson R. E., Heideman W. (2013). Sox9b is required for epicardium formation and plays a role in TCDD-induced heart malformation in zebrafish. *Molecular Pharmacology*.

[CIT0055] Huggett R. J., Bender M. E., Unger M. A., Dickson K. L., Maki A. W., Brungs W. A. (1987). Polynuclear aromatic hydrocarbons in the Elizabeth River, Virginia, USA. *Fate and effects of sediment-bound chemicals in aquatic systems*.

[CIT0056] Huggett R. J., van Veld P. A., Smith C. L., Hargis, Jr. W. J., Vogelbein W. K., Weeks B. A., Burton G. A., Jr. (1992). The effects of contaminated sediments in the Elizabeth River. *Sediment toxicity assessment*.

[CIT0057] Incardona J., Day H. L., Collier T. K., Scholz N. L. (2006). Developmental toxicity of 4-ring polycyclic aromatic hydrocarbons in zebrafish is differentially dependent on AH receptor isoforms and hepatic cytochrome P4501A metabolism. *Toxicology and Applied Pharmacology*.

[CIT0058] Incardona J. P., Carls M. G., Teraoka H., Sloan C. A., Collier T. K., Scholz N. L. (2005). Aryl hydrocarbon receptor-independent toxicity of weathered crude oil during fish development. *Environmental Health Perspectives*.

[CIT0059] Incardona J. P., Collier T. K., Scholz N. L. (2004). Defects in cardiac function precede morphological abnormalities in fish embryos exposed to polycyclic aromatic hydrocarbons. *Toxicology and Applied Pharmacology*.

[CIT0060] James M. O., Little P. J. (1983). Modification of benzo(a)pyrene metabolism in hepatic microsomes from untreated and induced rats by imidazole derivatives which inhibit monooxygenase activity and enhance epoxide hydrolase activity. *Drug Metabolism and Disposition*.

[CIT0061] Jayasundara N., Van Tiem Garner L., Meyer J. N., Erwin K. N., Di Giulio R. T. (2015). AHR2-mediated transcriptomic responses underlying the synergistic cardiac developmental toxicity of PAHs. *Toxicological Sciences*.

[CIT0062] Jung D., Cho Y., Collins L. B., Swenberg J. A., Di Giulio R. T. (2009a). Effects of benzo[a]pyrene on mitochondrial and nuclear DNA damage in Atlantic killifish (*Fundulus heteroclitus*) from a creosote-contaminated and reference site. *Aquatic Toxicology*.

[CIT0063] Jung D., Cho Y., Meyer J. N., Di Giulio R. T. (2009b). The long amplicon quantitative PCR for DNA damage assay as a sensitive method of assessing DNA damage in the environmental model, Atlantic killifish (*Fundulus heteroclitus*). *Comparative Biochemistry and Physiology Part C: Toxicology & Pharmacology*.

[CIT0064] Jung D., Di Giulio R. T. (2010). Identification of mitochondrial cytochrome P450 induced in response to polycyclic aromatic hydrocarbons in the mummichog (*Fundulus heteroclitus*). *Comparative Biochemistry and Physiology Part C: Toxicology & Pharmacology*.

[CIT0065] Kaplan B. L. F., Sulentic C. E. W., Holsapple M. P., Kaminski N. E., Klaassen C. D. (2013). Toxic responses of the immune system. *Casarett and Doull’s toxicology: The basic science of toxicology*.

[CIT0066] Karnaky K. J., Degnan K. J., Zadunaisky J. A. (1977). Chloride transport across isolated opercular epithelium of killifish: A membrane rich in chloride cells. *Science*.

[CIT0067] Karnaky K. J., Kinter L. B., Kinter W. B., Stirling C. E. (1976). Teleost chloride cell. 2. Autoradiographic localizations of gill Na,K-ATPase in kilifish (*Fundulus heteroclitus*) adapted to low and high salinity environments. *Journal of Cell Biology*.

[CIT0068] Kaspar J. W., Niture S. K., Jaiswal A. K. (2009). Nrf2: INrf2 (Keap1) signaling in oxidative stress. *Free Radical Biology and Medicine*.

[CIT0069] Kelly-Reay K., Weeks-Perkins B. A. (1994). Determination of the macrophage chemiluminescent response in *Fundulus heteroclitus* as a function of pollution stress. *Fish & Shellfish Immunology*.

[CIT0070] Kinnison M. T., Hairston N. G. (2007). Eco-evolutionary conservation biology: Contemporary evolution and the dynamics of persistence. *Functional Ecology*.

[CIT0071] Kleinow K. M., James M. O., Tong Z., Venugopalan C. S. (1998). Bioavailability and biotransformation of benzo(a)pyrene in an isolated perfused in situ catfish intestinal preparation. *Environmental Health Perspectives*.

[CIT0072] Kneib R. T. (1982). The effects of predation by wading birds (Ardeidae) and blue crabs (*Callinectes sapidus*) on the population size structure of the common mummichog, *Fundulus heteroclitus*. *Estuarine, Coastal and Shelf Science*.

[CIT0073] Kneib R. T. (1986). The role of *Fundulus heteroclitus* in salt-marsh trophic dynamics. *American Zoologist*.

[CIT0074] Kneib R. T., Stiven A. E. (1978). Growth, reproduction, and feeding of *Fundulus heteroclitus* (L.) on a North Carolina salt marsh. *Journal of Experimental Marine Biology and Ecology*.

[CIT0075] Kraemer L. D., Schulte P. M. (2004). Prior PCB exposure suppresses hypoxia-induced up-regulation of glycolytic enzymes in *Fundulus heteroclitus*. *Comparative Biochemistry and Physiology Part C: Toxicology & Pharmacology*.

[CIT0076] Lenormand T., Bourguet D., Guillemaud T., Raymond M. (1999). Tracking the evolution of insecticide resistance in the mosquito *Culex pipiens*. *Nature*.

[CIT0077] Lotrich V. A. (1975). Summer home range and movements of *Fundulus heteroclitus* (Pisces: Cyprinodontidae) in a Tidal Creek. *Ecology*.

[CIT0078] Luch A. (2005). *The carcinogenic effects of polycyclic aromatic hydrocarbons*.

[CIT0079] Marshall W. S., Bryson S. E., Midelfart A., Hamilton W. F. (1995). Low-conductance anion channel activated by cAMP in teleost Cl–secreting cells. *American Journal of Physiology—Regulatory, Integrative, and Comparative Physiology*.

[CIT0080] Matson C. W., Clark B. W., Jenny M. J., Fleming C. R., Hahn M. E., Di Giulio R. T. (2008). Development of the morpholino gene knockdown technique in *Fundulus heteroclitus*: A tool for studying molecular mechanisms in an established environmental model. *Aquatic Toxicology*.

[CIT0081] McMahon K. W., Johnson B. J., Ambrose W. G. (2005). Diet and movement of the killifish, *Fundulus heteroclitus*, in a Maine salt marsh assessed using gut contents and stable isotope analyses. *Estuaries*.

[CIT0082] McMillan A. M., Bagley M. J., Jackson S. A., Nacci D. E. (2006). Genetic diversity and structure of an estuarine fish (*Fundulus heteroclitus*) indigenous to sites associated with a highly contaminated urban harbor. *Ecotoxicology*.

[CIT0083] Merrill E. G., Wade T. L. (1985). Carbonized coal products as a source of aromatic hydrocarbons to sediments from a highly industrialized estuary. *Environmental Science & Technology*.

[CIT0084] Meyer J., Di Giulio R. (2002). Patterns of heritability of decreased EROD activity and resistance to PCB 126-induced teratogenesis in laboratory-reared offspring of killifish (*Fundulus heteroclitus*) from a creosote-contaminated site in the Elizabeth River, VA, USA. *Marine Environmental Research*.

[CIT0085] Meyer J. N., Di Giulio R. T. (2003). Heritable adaptation and fitness costs in killifish (*Fundulus heteroclitus*) inhabiting a polluted estuary. *Ecological Applications*.

[CIT0086] Meyer J. N., Nacci D. E., Di Giulio R. T. (2002). Cytochrome P4501A (CYP1A) in killifish (*Fundulus heteroclitus*): Heritability of altered expression and relationship to survival in contaminated sediments. *Toxicological Sciences*.

[CIT0087] Meyer J. N., Smith J. D., Winston G. W., Di Giulio R. T. (2003a). Antioxidant defenses in killifish (*Fundulus heteroclitus*) exposed to contaminated sediments and model prooxidants: Short-term and heritable responses. *Aquatic Toxicology*.

[CIT0088] Meyer J. N., Volz D. C., Freedman J. H., Di Giulio R. T. D. (2005). Differential display of hepatic mRNA from killifish (*Fundulus heteroclitus*) inhabiting a Superfund estuary. *Aquatic Toxicology*.

[CIT0089] Meyer J. N., Wassenberg D. M., Karchner S. I., Hahn M. E., Di Giulio R. T. (2003b). Expression and inducibility of aryl hydrocarbon receptor pathway genes in wild-caught killifish (*Fundulus heteroclitus*) with different contaminant-exposure histories. *Environmental Toxicology and Chemistry/SETAC*.

[CIT0090] Monteverdi G. H., Di Giulio R. T. (2000). Oocytic accumulation and tissue distribution of 2,3,7,8-tetrachlorodibenzo-*p*-dioxin and benzo[a]pyrene in gravid *Fundulus heteroclitus*. *Environmental Toxicology and Chemistry/SETAC*.

[CIT0091] Mulvey M., Newman M. C., Vogelbein W., Unger M. A. (2002). Genetic structure of *Fundulus heteroclitus* from PAH-contaminated and neighboring sites in the Elizabeth and York Rivers. *Aquatic Toxicology*.

[CIT0092] Mulvey M., Newman M. C., Vogelbein W. K., Unger M. A., Ownby D. R. (2003). Genetic structure and mtDNA diversity of *Fundulus heteroclitus* populations from polycyclic aromatic hydrocarbon-contaminated sites. *Environmental Toxicology and Chemistry/SETAC*.

[CIT0093] Nacci D., Champlin D., Jayaraman S. (2010). Adaptation of the estuarine fish *Fundulus heteroclitus* (Atlantic killifish) to polychlorinated biphenyls (PCBs). *Estuaries Coasts*.

[CIT0094] Nacci D., Coiro L., Champlin D., Jayaraman S., McKinney R., Gleason T. R., Munns, Jr. W. R., Specker J. L., Cooper K. R. (1999). Adaptations of wild populations of the estuarine fish *Fundulus heteroclitus* to persistent environmental contaminants. *Marine Biology*.

[CIT0095] Nacci D. E., Kohan M., Pelletier M., George E. (2002). Effects of benzo[a]pyrene exposure on a fish population resistant to the toxic effects of dioxin-like compounds. *Aquatic Toxicology*.

[CIT0096] Nemerson D. M., Able K. W. (2003). Spatial and temporal patterns in the distribution and feeding habits of *Morone saxatilis* in marsh creeks of Delaware Bay, USA. *Fisheries Management and Ecology*.

[CIT0097] Nicholas D. (1973). *Wood deterioration and its prevention by preservative treatments degradation and protection of wood*.

[CIT0098] Nordlie F. G. (2006). Physicochemical environments and tolerances of cyprinodontoid fishes found in estuaries and salt marshes of eastern North America. *Reviews in Fish Biology and Fisheries*.

[CIT0099] Notch E. G., Chapline C., Flynn E., Lameyer T., Lowell A., Sato D., Shaw J. R., Stanton B. A. (2012). Mitogen activated protein kinase 14-1 regulates serum glucocorticoid kinase 1 during seawater acclimation in Atlantic killifish, *Fundulus heteroclitus*. *Comparative Biochemistry and Physiology Part A: Molecular & Integrative Physiology*.

[CIT0100] Notch E. G., Shaw J. R., Coutermarsh B. A., Dzioba M., Stanton B. A., Laudet V. (2011). Morpholino gene knockdown in adult *Fundulus heteroclitus*: Role of SGK1 in seawater acclimation. *PLoS ONE*.

[CIT0101] Oesch F. (1988). Antimutagenesis by shift in monooxygenase isoenzymes and induction of epoxide hydrolase. *Mutation Research*.

[CIT0102] Oleksiak M. F. (2008). Changes in gene expression due to chronic exposure to environmental pollutants. *Aquatic Toxicology*.

[CIT0103] Oleksiak M. F., Churchill G. A., Crawford D. L. (2002). Variation in gene expression within and among natural populations. *Nature Genetics*.

[CIT0104] Oleksiak M. F., Roach J. L., Crawford D. L. (2005). Natural variation in cardiac metabolism and gene expression in *Fundulus heteroclitus*. *Nature Genetics*.

[CIT0105] Ownby D. R., Newman M. C., Mulvey M., Vogelbein W. K., Unger M. A., Arzayus L. F. (2002). Fish (*Fundulus heteroclitus*) populations with different exposure histories differ in tolerance of creosote-contaminated sediments. *Environmental Toxicology and Chemistry/SETAC*.

[CIT0106] Oziolor E. M., Bigorgne E., Aguilar L., Usenko S., Matson C. W. (2014). Evolved resistance to PCB- and PAH-induced cardiac teratogenesis, and reduced CYP1A activity in Gulf killifish (*Fundulus grandis*) populations from the Houston Ship Channel, Texas. *Aquatic Toxicology*.

[CIT0107] Peltonen K., Dipple A. (1995). Polycyclic aromatic hydrocarbons: Chemistry of DNA adduct formation. *Journal of Occupational and Environmental Medicine*.

[CIT0108] Perera F. P., Tang D. L., Wang S., Vishnevetsky J., Zhang B. Z., Diaz D., Camann D., Rauh V. (2012). Prenatal polycyclic aromatic hydrocarbon (PAH) exposure and child behavior at age 6-7 years. *Environmental Health Perspectives*.

[CIT0109] Peterson J. S. K., Bain L. J. (2004). Differential gene expression in anthracene-exposed mummichogs (*Fundulus heteroclitus*). *Aquatic Toxicology*.

[CIT0110] Peterson R. E., Theobald H. M., Kimmel G. L. (1993). Developmental and reproductive toxicity of dioxins and related compounds: Cross-species comparisons. *Critical Reviews in Toxicology*.

[CIT0111] Place A. R., Powers D. A. (1979). Genetic variation and relative catalytic efficiencies: Lactate dehydrogenase B allozymes of *Fundulus heteroclitus*.. *Proceedings of the National Academy of Sciences of the United States of America*.

[CIT0112] Post W. (2008). Food exploitation patterns in an assembly of estuarine herons. *Waterbirds*.

[CIT0113] Prince R., Cooper K. R. (1995). Comparisons of the effects of 2,3,7,8-tetrachlorodibenzo-p-dioxin on chemically impacted and nonimpacted subpopulations of *Fundulus heteroclitus* .1. TCDD toxicity. *EnvironToxicol Chemical*.

[CIT0114] Proestou D. A., Flight P., Champlin D., Nacci D. (2014). Targeted approach to identify genetic loci associated with evolved dioxin tolerance in Atlantic killifish (*Fundulus heteroclitus*). *BMC Evolution Biology*.

[CIT0115] Reynaud S., Deschaux P. (2006). The effects of polycyclic aromatic hydrocarbons on the immune system of fish: A review. *Aquatic Toxicology*.

[CIT0116] Ribeiro R., Lopes I. (2013). Contaminant driven genetic erosion and associated hypotheses on alleles loss, reduced population growth rate and increased susceptibility to future stressors: An essay. *Ecotoxicology*.

[CIT0117] Roark S. A., Nacci D., Coiro L., Champlin D., Guttman S. I. (2005). Population genetic structure of a nonmigratory estuarine fish (*Fundulus heteroclitus*) across a strong gradient of polychlorinated biphenyl contamination. *Environmental Toxicology and Chemistry*.

[CIT0118] Roberts M. H., Sved D. W., Felton S. P. (1987). Temporal changes in AHH and SOD activities in feral spot from the Elizabeth River, a polluted sub-estuary. *Marine Environmental Research*.

[CIT0119] Roling J. A., Bain L. J., Baldwin W. S. (2004). Differential gene expression in mummichogs (*Fundulus heteroclitus*) following treatment with pyrene: Comparison to a creosote contaminated site. *Marine Environmental Research*.

[CIT0120] Roling J. A., Bain L. J., Gardea-Torresdey J., Bader J., Baldwin W. S. (2006). Hexavalent chromium reduces larval growth and alters gene expression in mummichog (*Fundulus heteroclitus*). *Environmental Toxicology and Chemistry/SETAC*.

[CIT0121] Roling J. A., Bain L. J., Gardea-Torresdey J., Key P. B., Baldwin W. S. (2007). Using mummichog (*Fundulus heteroclitus*) arrays to monitor the effectiveness of remediation at a superfund site in Charleston, South Carolina, USA. *Environmental Toxicology and Chemistry/SETAC*.

[CIT0122] Roling J. A., Baldwin W. S. (2006). Alterations in hepatic gene expression by trivalent chromium in *Fundulus heteroclitus*. *Marine Environmental Research*.

[CIT0123] Rose W. L., French B. L., Reichert W. L., Faisal M. (2000). DNA adducts in hematopoietic tissues and blood of the mummichog (*Fundulus heteroclitus*) from a creosote-contaminated site in the Elizabeth River, Virginia. *Marine Environmental Research*.

[CIT0124] Roy N. K., Courtenay S., Maxwell G., Yuan Z. P., Chambers R. C., Wirgin I. (2002). Cytochrome P4501A1 is induced by PCB 77 and benzo[a]pyrene treatment but not by exposure to the Hudson River environment in Atlantic tomcod (Microgadus tomcod) post-yolk sac larvae. *Biomarkers*.

[CIT0125] Schneider A. J., Branam A. M., Peterson R. E. (2014). Intersection of AHR and Wnt signaling in development, health, and disease. *International Journal of Molecular Sciences*.

[CIT0126] Schulte P. M. (2001). Environmental adaptations as windows on molecular evolution. *Comparative Biochemistry and Physiology Part B: Biochemistry and Molecular Biology*.

[CIT0127] Schulte P. M., Glemet H. C., Fiebig A. A., Powers D. A. (2000). Adaptive variation in lactate dehydrogenase-B gene expression: Role of a stress-responsive regulatory element. *Proceedings of the National Academy of Sciences of the United States of America*.

[CIT0128] Schulte P. M., GomezChiarri M., Powers D. A. (1997). Structural and functional differences in the promoter and 5’ flanking region of Ldh-B within and between populations of the teleost *Fundulus heteroclitus*. *Genetics*.

[CIT0129] Shute J. R., Lee D. S., Gilbert C. R., Hocutt V. H., Jenkins R. E., McAllister D. C., Stauffer J. R. (1980). *Fundulus heteroclitus* Linnaeus. *Atlas of North American freshwater fishes*.

[CIT0130] Singer T. D., Tucker S. J., Marshall W. S., Higgins C. F. (1998). A divergent CFTR homologue: Highly regulated salt transport in the euryhaline teleost F-heteroclitus. *American Journal of Physiology—Cell Physiology*.

[CIT0131] Skinner M. A., Courtenay S. C., Parker W. R., Curry R. A. (2005). Site fidelity of mummichogs (*Fundulus heteroclitus*) in an Atlantic Canadian estuary. *Water Qualitative Researcher Journal Canada*.

[CIT0132] Smith K. J., Able K. W. (2003). Dissolved oxygen dynamics in salt marsh pools and its potential impacts on fish assemblages. *Marine Ecology Progress Series*.

[CIT0133] Sorrentino C., Roy N. K., Chambers R. C., Courtenay S. C., Wirgin I. (2004). B[a]P-DNA binding in early life-stages of Atlantic tomcod: Population differences and chromium modulation. *Marine Environmental Research*.

[CIT0134] Stegeman J. J., James M. O. (1985). Individual variation in patterns of benzo[a]pyrene metabolism in the marine fish scup (*Stenotomus chrysops*). *Marine Environmental Research*.

[CIT0135] Stierhoff K. L., Targett T. E., Grecay P. A. (2003). Hypoxia tolerance of the mummichog: The role of access to the water surface. *Journal of Fish Biology*.

[CIT0136] Stine C. B., Smith D. L., Vogelbein W. K., Harshbarger J. C., Gudla P. R., Lipsky M. M., Kane A. S. (2004). Morphometry of hepatic neoplasms and altered foci in the mummichog, *Fundulus heteroclitus*. *Toxicologic Pathology*.

[CIT0137] Sved D. W., Roberts M. H., VanVeld P. A. (1997). Toxicity of sediments contaminated with fractions of creosote. *Water Research*.

[CIT0138] Taylor M. H., Leach G. J., Dimichele L., Levitan W. M., Jacob W. F. (1979). Lunar spawning cycle in the mummichog, *Fundulus heteroclitus* (Pisces: Cyprinodontidae). *Copeia*.

[CIT0139] Teo S. L. H., Able K. W. (2003a). Growth and production of the mummichog (*Fundulus heteroclitus*) in a restored salt marsh. *Estuaries*.

[CIT0140] Teo S. L. H., Able K. W. (2003b). Habitat use and movement of the mummichog (*Fundulus heteroclitus*) in a restored salt marsh. *Estuaries*.

[CIT0141] Teraoka H., Okuno Y., Nijoukubo D., Yamakoshi A., Peterson R. E., Stegeman J. J., Kitazawa T., Hiraga T., Kubota A. (2014). Involvement of COX2-thromboxane pathway in TCDD-induced precardiac edema in developing zebrafish. *Aquatic Toxicology*.

[CIT0142] Thiyagarajah A., Zwerner D. E., Hargis W. J. J. (1989). Renal lesions in estuarine fishes collected from the Elizabeth River, Virginia, USA. *Journal of Environmental Pathology, Toxicology and Oncology : Official Organ of the International Society for Environmental Toxicology and Cancer*.

[CIT0143] Timme-Laragy A. R., Meyer J. N., Waterland R. A., Di Giulio R. T. (2005). Analysis of CpG methylation in the killifish CYP1A promoter. *Comparative Biochemistry and Physiology Part C: Toxicology & Pharmacology*.

[CIT0144] Timme-Laragy A. R., Van Tiem L. A., Linney E. A., Di Giulio R. T. (2009). Antioxidant responses and NRF2 in synergistic developmental toxicity of PAHs in zebrafish. *Toxicological Sciences*.

[CIT0145] U.S. Environmental Protection Agency (1994). *Chesapeake Bay basinwide toxics reduction strategy evaluation report*.

[CIT0146] U.S. Environmental Protection Agency (2010). *Development of a relative potency factor (RPF) approach for polycyclic aromatic hydrocarbon (PAH) mixtures (Draft)*.

[CIT0147] Van Den Berg M., Birnbaum L. S., Denison M., De Vito M., Farland W., Feeley M., Fiedler H., Hakansson H., Hanberg A., Haws L., Rose M., Safe S., Schrenk D., Tohyama C., Tritscher A., Tuomisto J., Tysklind M., Walker N., Peterson R. E. (2006). The 2005 World Health Organization reevaluation of human and mammalian toxic equivalency factors for dioxins and dioxin-like compounds. *Toxicological Sciences*.

[CIT0148] Van Metre P. C., Mahler B. J. (2005). Trends in hydrophobic organic contaminants in urban and reference lake sediments across the United States, 1970−2001. *Environmental Science & Technology*.

[CIT0149] Van Tiem L. A., Di Giulio R. T. (2011). AHR2 knockdown prevents PAH-mediated cardiac toxicity and XRE- and ARE-associated gene induction in zebrafish (*Danio rerio*). *Toxicology and Applied Pharmacology*.

[CIT0150] Van Veld P. A., Ko U. C., Vogelbein W. K., Westbrook D. J. (1991). Glutathione-s-transferase in intestine, liver and hepatic lesions of mummichog (*Fundulus heteroclitus*) from a creosote-contaminated environment. *Fish Physiology and Biochemistry*.

[CIT0151] Van Veld P. A., Nacci D., Giulio R. T. D., Hinton D. E. (2008). Toxicity resistance. *The toxicology of fishes*.

[CIT0152] Van Veld P. A., Westbrook D. J. (1995). Evidence for depression of cytochrome P4501A in a population of chemically resistant mummichog (*Fundulus heteroclitus*). *Environmental Sciences*.

[CIT0153] Virginia State Water Control Board (1976). Virginia Water Quality Assessment Bulletin 526, 1976-305 (B) Report to EPA Administrator and Congress.

[CIT0154] Vogelbein W., Unger M. A. (2003). Final report to the Virginia Department of Environmental Quality. The Elizabeth River monitoring program 2001–2002: Association between mummichog liver histopathology and sediment chemical contamination.

[CIT0155] Vogelbein W., Unger M. A. (2008). Final Report to the Virginia Department of Environmental Quality.. The Elizabeth River monitoring program 2006-2007: Association between mummichog liver histopathology and sediment chemical contamination.

[CIT0156] Vogelbein W. K., Fournie J. W., Vanveld P. A., Huggett R. J. (1990). Hepatic neoplasms in the mummichog *Fundulus heteroclitus* from a creosote-contaminated site. *Cancer Research*.

[CIT0157] Walker S. E., Dickhut R. M. (2001). Sources of PAHs to sediments of the Elizabeth River, VA. *Soil Sediment Contamination*.

[CIT0158] Walker S. E., Dickhut R. M., Chisholm-Brause C. (2004). Polycyclic aromatic hydrocarbons in a highly industrialized urban estuary: Inventories and trends. *Environmental Toxicology and Chemistry/SETAC*.

[CIT0159] Walker S. E., Dickhut R. M., Chisholm-Brause C., Sylva S., Reddy C. M. (2005). Molecular and isotopic identification of PAH sources in a highly industrialized urban estuary. *Organic Geochemistry*.

[CIT0160] Wannamaker C. M., Rice J. A. (2000). Effects of hypoxia on movements and behavior of selected estuarine organisms from the southeastern United States. *Journal of Experimental Marine Biology and Ecology*.

[CIT0161] Wassenberg D. M., Di Giulio R. T. (2004a). Synergistic embryotoxicity of polycyclic aromatic hydrocarbon aryl hydrocarbon receptor agonists with cytochrome P4501A inhibitors in *Fundulus heteroclitus*. *Environmental Health Perspectives*.

[CIT0162] Wassenberg D. M., Di Giulio R. T. (2004b). Teratogenesis in *Fundulus heteroclitus* embryos exposed to a creosote-contaminated sediment extract and CYP1A inhibitors. *Marine Environmental Research*.

[CIT0163] Wassenberg D. M., Nerlinger A. L., Battle L. P., Di Giulio R. T. (2005). Effects of the polycyclic aromatic hydrocarbon heterocycles, carbazole and dibenzothiophene, on in vivo and in vitro CYP1A activity and polycyclic aromatic hydrocarbon-derived embryonic deformities. *Environmental Toxicology and Chemistry/SETAC*.

[CIT0164] Weeks B. A., Warinner J. E. (1984). Effects of toxic chemicals on macrophage phagocytosis in two estuarine fishes. *Marine Environmental Research*.

[CIT0165] Weeks B. A., Warinner J. E., Mathews E. S. (1988). Influence of toxicants on phagocytosis, pinocytosis and melanin accumulation by fish macrophages. *Aquatic Toxicology*.

[CIT0166] Weis J. S., Weis P., Heber M., Vaidya S. (1981). Methylmercury tolerance of killifish (*Fundulus heteroclitus*) embryos from a polluted vs non-polluted environment. *Marine Biologic*.

[CIT0167] Whitehead A., Crawford D. L. (2006a). Neutral and adaptive variation in gene expression. *Proceedings of the National Academy of Sciences*.

[CIT0168] Whitehead A., Crawford D. L. (2006b). Variation within and among species in gene expression: Raw material for evolution. *Molecular Ecology*.

[CIT0169] Whitehead A., Pilcher W., Champlin D., Nacci D. (2012). Common mechanism underlies repeated evolution of extreme pollution tolerance. *Proceedings Royal Society B Biological Sciences*.

[CIT0170] Whitehead A., Triant D. A., Champlin D., Nacci D. (2010). Comparative transcriptomics implicates mechanisms of evolved pollution tolerance in a killifish population. *Molecular Ecology*.

[CIT0171] Williams C. A. H. (1994). Toxicity resistance in mummichog (*Fundulus heteroclitus*) from a chemically contaminated environment. Master of arts thesis.

[CIT0172] Williams L. M., Oleksiak M. F. (2008). Signatures of selection in natural populations adapted to chronic pollution. *BMC Evolutionary Biology*.

[CIT0173] Wills L. P., Jung D., Koehrn K., Zhu S. Q., Willett K. L., Hinton D. E., Di Giulio R. T. (2010a). Comparative chronic liver toxicity of benzo[a]pyrene in two populations of the Atlantic killifish (*Fundulus heteroclitus*) with different exposure histories. *Environmental Health Perspectives*.

[CIT0174] Wills L. P., Matson C. W., Landon C. D., Di Giulio R. T. (2010b). Characterization of the recalcitrant CYP1 phenotype found in Atlantic killifish (*Fundulus heteroclitus*) inhabiting a Superfund site on the Elizabeth River, VA. *Aquatic Toxicology*.

[CIT0175] Wills L. P., Zhu S. Q., Willett K. L., Di Giulio R. T. (2009). Effect of CYP1A inhibition on the biotransformation of benzo[a]pyrene in two populations of *Fundulus heteroclitus* with different exposure histories. *Aquatic Toxicology*.

[CIT0176] Winn R. N., Vanbeneden R. J., Burkhart J. G. (1995). Transfer, methylation and spontaneous mutation frequency of ΦX174am3cs70 sequences in medaka (*Oryzias latipes*) and mummichog (*Fundulus heteroclitus*): Implications for gene transfer and environmental mutagenesis in aquatic species. *Marine Environmental Research*.

[CIT0177] Wirgin I., Waldman J. R. (2004). Resistance to contaminants in North American fish populations. *Mutation Research*.

[CIT0178] Wondji C. S., Irving H., Morgan J., Lobo N. F., Collins F. H., Hunt R. H., Coetzee M., Hemingway J., Ranson H. (2008). Two duplicated P450 genes are associated with pyrethroid resistance in *Anopheles funestus*, a major malaria vector. *Genome Research*.

[CIT0179] Wood C. M., Marshall W. S. (1994). Ion balance, acid–base regulation, and chloride cell function in the common killifish, *Fundulus heteroclitus*: A euryhaline estuarine teleost. *Estuaries*.

[CIT0180] Wu B., Zhang R., Cheng S. P., Ford T., Li A. M., Zhang X. X. (2011). Risk assessment of polycyclic aromatic hydrocarbons in aquatic ecosystems. *Ecotoxicology*.

[CIT0181] Xie L. T., Klerks P. L. (2003). Responses to selection for cadmium resistance in the least killifish, Heterandria formosa. *Environmental Toxicology and Chemistry/SETAC*.

[CIT0182] Yarsinke A. W. (2007). *The Elizabeth River*.

[CIT0183] Yozzo D. J., Hester K. I., Smith D. E. (1994). Abundance and spawning site utilization of *Fundulus heteroclitus* at the Virginia Coast Reserve. *Virginia Journal of Science*.

